# Prevalence of fecal-detected gastrointestinal parasites in cattle, sheep, and goats across sub-Saharan Africa: a systematic evidence map and meta-analysis

**DOI:** 10.3389/fvets.2026.1984505

**Published:** 2026-10-15

**Authors:** Christian A. Bateki, Simon Tielkes, Eva Schlecht

**Affiliations:** 1 Section Animal Husbandry in the Tropics and Subtropics, University of Kassel and Georg-August-Universität Göttingen, Witzenhausen, Germany; 2 German Institute for Tropical and Subtropical Agriculture (DITSL) GmbH, Witzenhausen, Germany

**Keywords:** animal health surveillance, coprological diagnosis, livestock-based sustainable intensification, one health, parasite control, prevalence epidemiology, ruminants

## Abstract

**Introduction:**

Gastrointestinal parasites (GIPs) are a major constraint to the health and productive performance of ruminant livestock across sub-Saharan Africa (SSA). Yet, evidence of their occurrence in cattle, sheep, and goats (CSG) has not yet been synthesized at regional scale. This systematic evidence map and meta-analysis estimated the prevalence of fecal-detected GIP infection, described reported parasite taxa, and identified evidence gaps relevant to surveillance and control.

**Methods:**

Studies from countries in SSA reporting fecal-based GIP prevalence in CSG and published between 2015 and 2024 were identified via a systematic literature search conducted between April and July 2025. The pooled prevalence was estimated using random-effects meta-analysis of proportions, with subgroup analyses by host species, geographic regions, sample source, and fecal analysis method.

**Results:**

111 studies from 18 countries, comprising 57,500 animals, were included. The pooled prevalence of infection with one or more GIP taxa in CSG was 59.1% (95% CI, 54.6–63.6; 95% PI, 14.7–95.6). Prevalence was higher in sheep (64.0%) and goats (63.3%) than in cattle (48.2%). Prevalence was highest in West (74.9%) and Central Africa (74.0%), followed by East Africa (55%), and lowest in Southern Africa (26.4%), although heterogeneity was very high (*I*^2^ = 99.2%). *Strongyloides* and *Fasciola* were the most frequently reported genera, while *Haemonchus contortus* and *Fasciola gigantica* were the most frequently reported species among records with species-level identification. Evidence was concentrated in Ethiopia and Nigeria, and most studies relied on routine coprological methods with limited species-level resolution.

**Conclusions:**

This first SSA-wide evidence map identified major geographic and diagnostic surveillance gaps and points to strongyle-type nematodes in small ruminants and *Fasciola* spp. in cattle as priority targets for improved surveillance and integrated parasite management.

## 1 Introduction

Sub-Saharan Africa (SSA) is currently home to an estimated 1.1 billion heads of cattle, sheep, and goats (CSG), which make up about 27% of the global CSG population (1). These CSG utilize grasses and other fiber-rich feed resources to produce meat and milk which are crucial for the food and nutritional security of millions of Africans (2, 3). They provide draft power (i.e., cattle) and manure which serve as crucial inputs for smallholder agriculture in the region (4); they also contribute significantly to the livelihoods of millions of people in SSA by providing employment and accounting for up to 75% of household incomes in rural areas (5). In addition, CSG serve as social capital and occupy a special place in the African culture with important roles in traditional ceremonies and rites where they are used as means of compensation or gifts (e.g., dowry) (6). Across SSA, changing socio-economic trends (e.g., higher per capita income and increasing urbanization) are driving the demand for livestock products upwards, with projections suggesting increases in the demand for animal-sourced proteins of up to 55% per person by 2050 (7). As such, many governments in the region are aiming to leverage CSG production to boost economic growth at national level.

Despite the multiple benefits that CSG production offers at farm and national levels in SSA, its full potential remains constrained by several factors related to nutrition and health of the animals, as well as climate change. Inadequate nutrition of CSG, often supplied with insufficient quantities of poor quality feeds (i.e., low in metabolizable energy and protein contents) is a primary factor limiting their performance (8). Many CSG farmers keep indigenous breeds which are well adapted to local production conditions but have a lower genetic potential for milk and meat production when compared to improved breeds (9, 10). In addition, the high prevalence of endemic diseases and high parasitic (i.e., both internal and external) burdens significantly hamper animal health by reducing feed conversion efficiency and thus productivity (11, 12). The impacts of these constraints are compounded by climate change, poor infrastructure (e.g., limited access to extension services, markets, and veterinary services), lack of financial resources, and inadequate institution support for CSG farmers (7, 13, 14). These issues must therefore be addressed if CSG production systems throughout SSA are to fulfill their full potential.

Animal diseases are a unique constraint to the development of CSG production in SSA because they hamper their productive and reproductive performance, causing significant animal losses. Previous estimates suggest that livestock keepers lose on average one animal per cattle sold and one animal for every two sheep or goats sold (15). While CSG are affected by different diseases, gastrointestinal parasites (GIPs) remain a major pathological cause of low efficiency and high mortality in these livestock (16, 17). Also, many GIPs cause zoonotic infections, posing a significant challenge to human health in the region, causing chronic health issues, as well as negatively affecting physical and mental development of exposed individuals (18, 19). In livestock, the losses caused by GIPs can be classified as direct or indirect (20). Direct losses result from acute GIP infections, that can often be recognized, treated, and thus mitigated. Conversely, indirect losses result from sub-clinical GIP infections with no obvious clinical signs, making them very difficult to recognize and avoid. Also, indirect losses are more salient in ruminants with poor nutritional status as is common in SSA, further compromising production efficiency and animal welfare (17).

While several studies have reported the prevalence of GIP infections in CSG in individual countries or local production systems across SSA (21–23), no study has systematically synthesized this evidence at the regional level. Such a synthesis is needed to characterize the available evidence base, identify the parasite groups most frequently reported, summarize the range of reported prevalence estimates, and to highlight geographical evidence gaps to inform disease prioritization frameworks and targeted control strategies in the region (24). Addressing these knowledge gaps will also provide valuable input to the Global Burden of Animal Diseases (GBADs) initiative, which aims to quantify the burden of animal diseases on human health, livelihoods, and wellbeing (25).

Meta-analysis can provide a robust statistical framework to quantitatively summarize the reported prevalence estimates of GIP infection in SSA based on published studies. When individual studies report a number of “positive cases” and a corresponding total sample size, meta-analysis of proportions is the recommended approach for deriving pooled prevalence estimates (26). Unlike conventional meta-analyses that summarize treatment effects by comparing intervention and control groups, meta-analysis of proportions estimates the total proportion related to the specific event across the included studies (27).

Therefore, the present study systematically reviewed published studies reporting the prevalence of GIP infections in CSG across SSA. Then using a meta-analysis of proportions, we summarized reported prevalence estimates across studies while identifying the parasite groups most frequently investigated and highlighted countries where epidemiological evidence remains limited. In this way, our study will contribute to evidence-based livestock health planning and support broader efforts toward sustainable livestock intensification and food and nutrition security in the region, in line with the United Nations' Sustainable Development Goals 2, 3, and 15.

## 2 Materials and methods

### 2.1 Ethics approval and consent to participate

The present study was exempt from the need for an Animal Care and Use Committee approval because it utilized data sourced from existing scientific literature.

### 2.2 Inclusion criteria

The inclusion criteria were formulated in line with the Condition, Context, Population (CoCoPop) framework for prevalence and incidence studies (28). The Condition was fecal detected GIP infection, Context was SSA, and Population was non-apparent sick CSG. The inclusion criteria were jointly defined by two reviewers (CAB and ST) and studies reporting cross-sectional, prevalence estimates were considered eligible. A study was included into the dataset if it (i) was conducted in SSA and published between 2015 and 2024 (i.e., last decade); (ii) was written in English; (iii) was accessible online as peer-reviewed article or grey literature; (iv) reported the prevalence of one or more GIPs in ruminant livestock based on fecal sample analyses; (v) focused on one or more of the three ruminant livestock species targeted (i.e., cattle, goats, and sheep); and (vi) clearly reported the total number of animals examined and the number that tested positive and/or negative for infection. Also, longitudinal (here only the first sampling was included) prevalence studies were included. Studies based on experimental infections, intervention trials, case reports, reviews, or modeling were excluded. No restrictions were imposed on sampling design or diagnostic technique, provided that fecal-based parasitological methods were used to confirm infection and prevalence could be calculated. Fecal-based parasitological methods were selected as the focus because they are widely used in SSA and field-applicable, facilitating the inclusion of evidence from countries with limited laboratory and analytical capacity.

### 2.3 Literature search and selection of studies

A systematic online literature search was conducted between April and July 2025 following the Preferred Reporting Items for Systematic Reviews and Meta-analyses (PRISMA) guidelines (Supplementary Table 1) to identify eligible studies for inclusion in the present study. The primary search was performed in Web of Science using combinations of keywords and Boolean operators related to parasite prevalence, ruminant species, and country names within sub-Saharan Africa. The main search query included terms related to prevalence (“prevalence,” “frequency,” “occurrence,” “incidence”), parasite groups (“gastrointestinal parasite^*^,” “nematodes,” “cestodes,” “trematodes,” “protozoa,” “Trichuris,” “Eimeria,” “Fasciola,” “gastrointestinal helminth^*^,” “strongyl^*^”), host species (“ruminant^*^,” “cattle,” “sheep,” “goats”), and all SSA countries, restricted to publications from 2015 to 2024. The full search strategy is provided in Supplementary material, page 4. Previous studies (29, 30) have shown that research outputs from SSA are under-represented in the major bibliographic databases. Consequently, we decided to search with Google Scholar because it indexes African Journals Online platform and other regional sources not consistently covered by Web of Science. As such, the same search query used for the Web of Science was rewritten (Supplementary material, page 4) and used to reduce the chance of missing relevant studies.

During the online literature search, titles and abstracts of all retrieved records were screened for eligibility. The full texts of potentially relevant studies were then downloaded for further assessment based on the inclusion criteria. The bibliographies of downloaded studies were examined to further identify studies eligible for inclusion into our dataset. The final literature search was completed on 31 July 2025.

### 2.4 Quality assessment of studies, data extraction, and data cleaning

Each eligible study was reviewed to assess its suitability for inclusion and to extract the data into a pre-designed Microsoft Excel^®^ spreadsheet. The variables extracted included general study details (e.g., publication year, country, specific sites, and design), weather data, ruminant species considered, sample sizes considered, number of animals that tested positive for GIPs, and the specific GIPs (species, genus, and family whenever available). Moreover, the quality of each study was first assessed based on the clarity of the reporting as well as the plausibility and coherence of the results reported in the tables and text. Plausibility checks of the results included verification that the (i) reported totals (i.e., animal numbers, prevalence) were internally consistent across tables and text for each ruminant species considered; and (ii) prevalence estimates could be clearly extracted. All studies with unresolved inconsistencies or insufficient reporting were excluded from further analysis. Next, a quality assessment using the Joanna Briggs Institute (JBI) checklist for studies reporting prevalence data was performed (28). Scores were calculated by assigning one point per “Yes” response across the eight applicable questions (Question 9 was considered inapplicable), then categorized as Low (7–8), Moderate (5–6), or High (0–4) risk of bias (Supplementary Table 2).

### 2.5 Statistical analysis

For each study in the dataset, the proportion of animals with fecal samples positive for one or more GIP taxa was used as the study-level prevalence estimate. All data analyses were carried out using R version 4.5.2 (R Foundation, Vienna, Austria), with the “meta” (31) and “metafor” (32) statistical packages. The overall prevalence of GIP infection across all species as well as the individual species was estimated with a 95% confidence interval using a random-effects model to account for the intra- and inter-study variances (26). The random effect model assumes that the true prevalence varies across studies and that the observed differences represent true variation in the underlying population. To address the skewness in the distribution of the dataset and stabilize the variance, the raw proportions (i.e., prevalence reported in each study) were normalized using the Freeman-Tukey double-arcsine transformation (33). Next, the random-effects models were estimated using the restricted maximum likelihood method (34) and the resulting proportions back-transformed following (26) to facilitate interpretation of the results. We quantified the inter-study heterogeneity using Cochran's Q (χ^2^) (35), *I*^2^ (36), and 95% prediction interval. Heterogeneity was deemed significant when *p* < 0.05 in the Cochran Q test and *I*^2^ > 50% (37).

Publication bias is a major issue affecting the validity of meta-analyses. Yet, commonly used methods for detecting it (e.g., visual inspection of funnel plots) were developed for meta-analyses of randomized control trials where the publication likelihood depends on the sample size and statistical significance of results (26). Meta-analyses of proportions such as the one undertaken in the present study, depend on observational rather than comparative studies, allowing studies reporting low or high prevalence an equal likelihood of publication. Therefore, we conducted a series of sensitivity analysis to assess the robustness of the pooled prevalence estimates. First, the influence of individual studies on the overall prevalence was assessed using a leave-one-out analysis. Then, diagnostic tests, including externally studentized residuals sorted according to the absolute values of the z-score were used to identify potentially outlying and influential studies (26). A Z-score cut-off point of 2.0 was used to identify studies with large externally studentized residuals. Similar to Zhang et al. (38), all studies flagged as influential were excluded sequentially, and the meta-analysis rerun to assess the robustness of the pooled prevalence estimates. Second, a risk-of-bias sensitivity analysis was performed based on the results of the JBI critical appraisal. The meta-analysis was repeated after excluding studies classified as having a high risk of bias to determine whether the pooled prevalence estimates were affected by the inclusion of studies with greater methodological concerns. Last, a subgroup analysis was performed by: geographic regions (i.e., West, Central, East, and Southern Africa), sample source (i.e., on-farm, abattoirs, and others, such as markets or mixed), and fecal diagnostic method (direct wet mount smear, flotation, flotation and sedimentation, immunodiagnostics, and sedimentation) to obtain within-subgroup levels of GIP infection.

## 3 Results

### 3.1 Characteristics and geographic distribution of evidence base

The literature search identified 507 study records, including 167 from Web of Science and 340 from Google Scholar (Figure 1).

**Figure 1:**
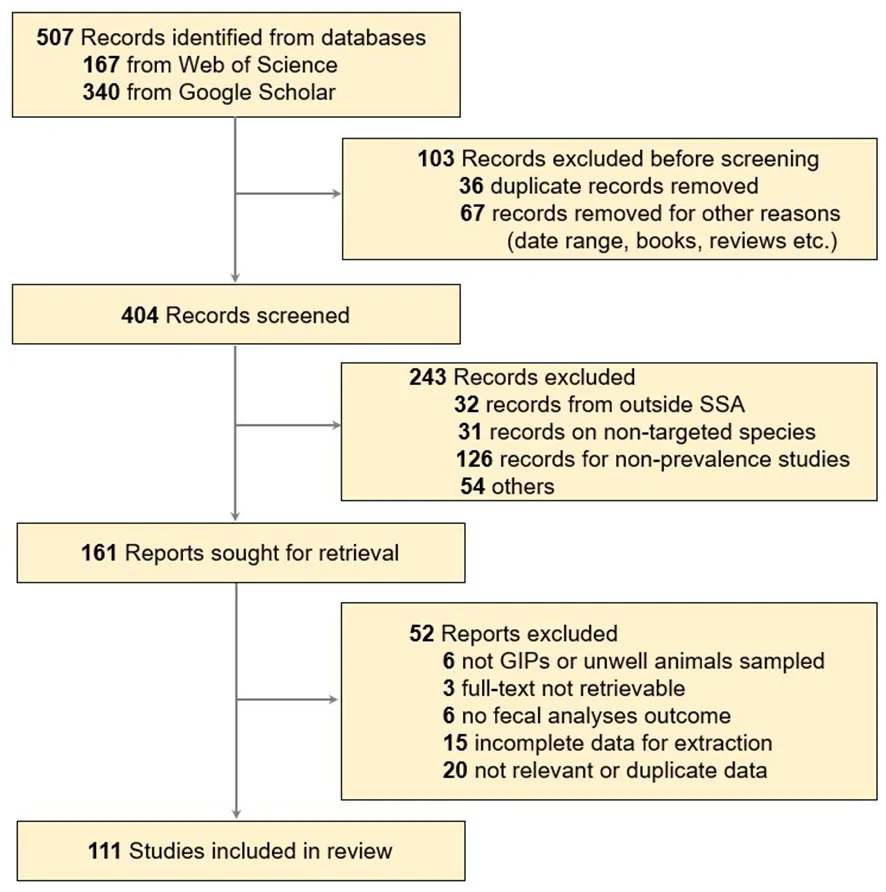
PRISMA flow diagram of the identification, screening, and inclusion of studies in the systematic evidence map and meta-analysis of fecal-detected gastrointestinal parasite infections in cattle, sheep, and goats in sub-Saharan Africa (SSA) from 2015 to 2024.

Automatic filtering excluded 103 records, including reviews, modelling studies, books, and other irrelevant records (e.g., wrong species or publication dates outside the predefined range). The titles and where necessary abstracts (e.g., when titles did not specify study location or animal species infected) of the remaining 404 records were subsequently screened. This initial screening led to the exclusion of 243 records, including studies that did not investigate the prevalence of GIP infections (e.g., indigenous knowledge, genomics, or external parasites), focused on non-targeted species, were conducted outside SSA, or were published in languages other than English. Of the remaining 161 study records, full texts of 158 studies were assessed for eligibility, as three full texts were not accessible despite attempts to contact the authors. Following full-text assessment, 52 studies were excluded for reasons shown in Figure 1. In total, 111 studies (including two doctoral theses), of which 25 included data collection spanning 2014–2015, 19 were conducted entirely before 2015, 46 entirely after 2015, and for 21 the year of data collection was not reported. The 111 studies comprised 57,500 animal fecal observations from 18 African countries across West, Central, East, and Southern Africa (Figure 2).

**Figure 2:**
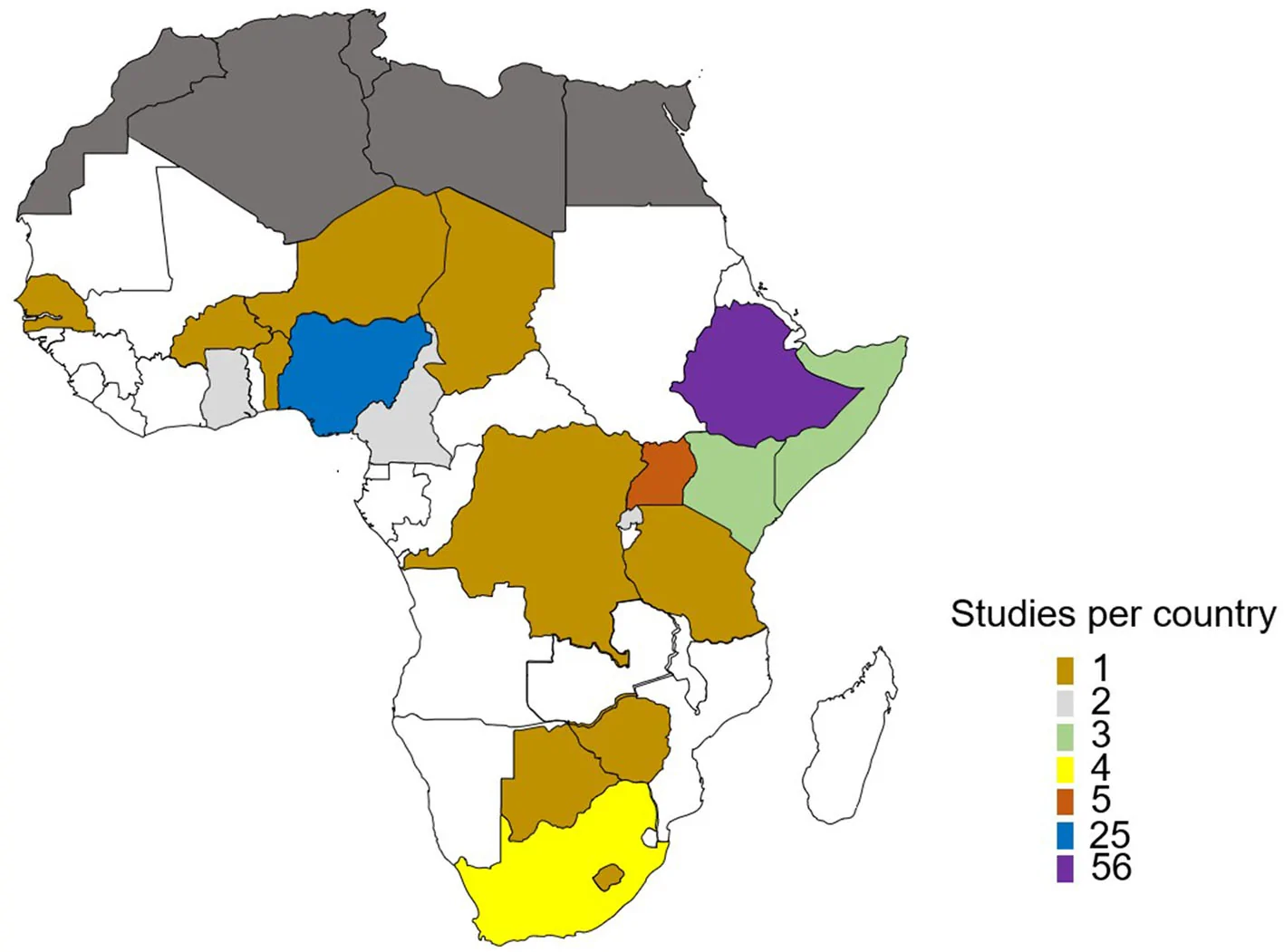
Geographical distribution of studies included in the systematic evidence map and meta-analysis of fecal-detected gastrointestinal parasite infections in cattle, sheep, and goats in sub-Saharan Africa. Colored countries indicate locations where one or more of the 111 included studies were conducted; white countries indicate that no eligible studies were identified; and dark-grey areas are outside the study region.

Forty studies focused exclusively on cattle, 21 on sheep, and 14 on goats, while the remaining studies investigated two (*n* = 30) or all three (*n* = 6) species. Across all studies, sample sizes ranged from 60 to 8,646 animals, yielding species-specific totals of 23,841 cattle, 20,109 sheep, and 11,752 goats. Two studies (39, 40) reported only aggregated sample sizes and prevalence estimates across species (i.e., CSG = 830 and sheep and goats = 968) without species-specific breakdowns and were thus excluded from the species-specific sample size totals.

Across the 111 studies, the flotation combined with sedimentation method was the most frequently used diagnostic approach (79 studies, 71.2%), followed by flotation only (19 studies, 17.1%) and sedimentation only (10 studies, 9.0%). The prevalence estimates of GIP infection across all studies ranged from 0.7% to 98.2%. Further characteristics of all 111 studies are presented in Supplementary Table 3.

### 3.2 Gastrointestinal parasite taxa identified across studies

Whereas all studies reported at least one parasite genus, most studies did not identify the GIPs to species level. Amongst records with species-level identification across CSG, *Haemonchus contortus* (*n* = 18) and *Fasciola gigantica* (*n* = 15) were those most frequently reported (Figure 3A). At genus level (Figure 3B), *Strongyloides* (*n* = 137), and *Fasciola* (*n* = 79) were most frequently reported.

**Figure 3:**
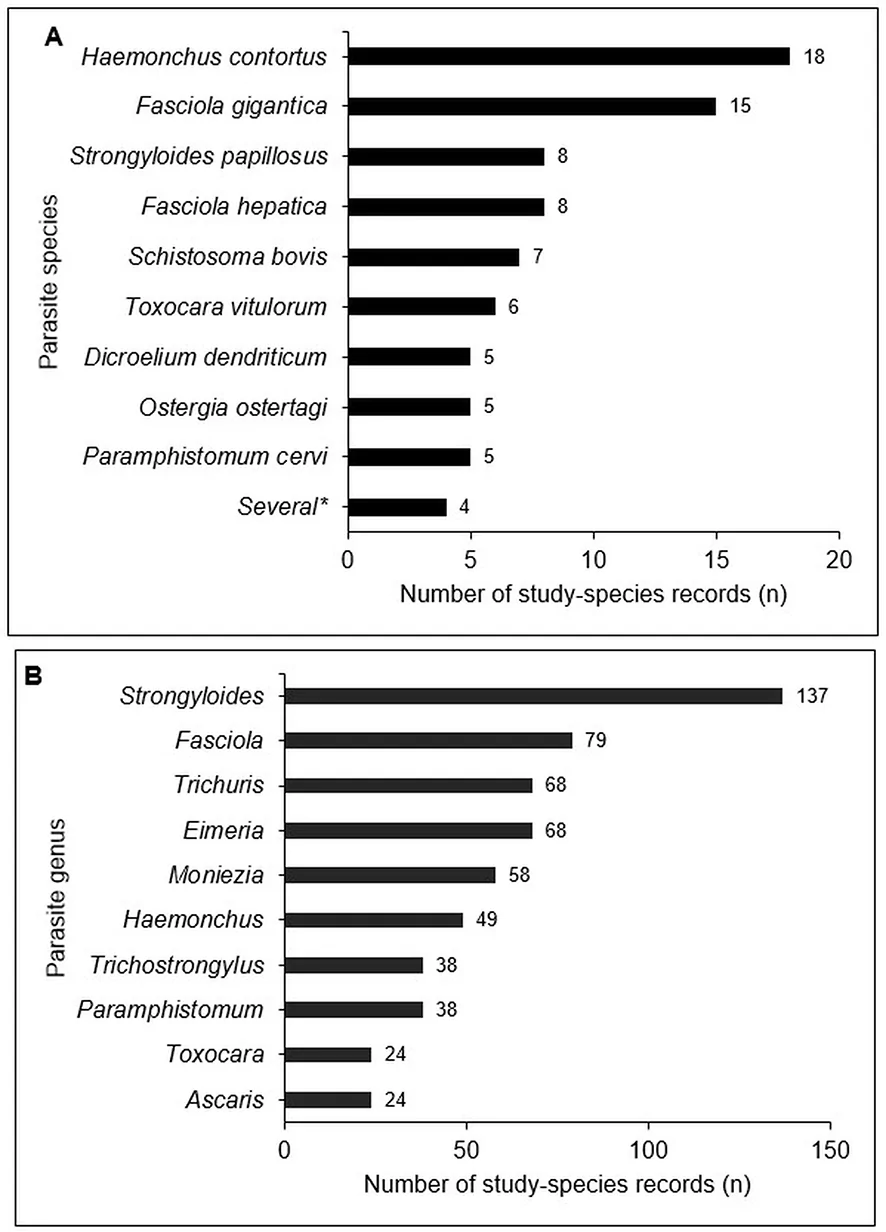
Frequency of gastrointestinal parasite taxa reported in included coprological study records from cattle, sheep, and goats in sub-Saharan Africa between 2015 and 2024. **(A)** The 10 most frequently reported parasite among records with species-level identification, Several* comprises *Bunostomum phlebotomum, Moniezia benedeni, and Moniezia expansa* **(B)** The 10 most frequently reported parasite genera identified in the fecal samples.

In cattle, *Fasciola gigantica* (*n* = 10) and *Schistosoma bovis* (*n* = 7) were the most frequently reported species; whereas *Fasciola* (*n* = 37), *Strongyloides* (*n* = 34), and *Eimeria* (*n* = 23) were most frequently reported genera (Supplementary Figure 1). In sheep, *Haemonchus contortus* (*n* = 6) and *Fasciola hepatica* (*n* = 3) were the most commonly reported species; while *Strongyloides* (*n* = 50), *Trichuris* (*n* = 22), as well as *Fasciola and Eimeria* (both *n* = 21) were most frequently reported at genus level (Supplementary Figure 1). In goats, *Haemonchus contortus* (*n* = 6) and *Strongyloides papillosus* (*n* = 4) were the most frequently reported species; while *Strongyloides* (*n* = 40), *Trichuris* (*n* = 23), and *Fasciola* (*n* = 21) were the most frequently reported genera (Supplementary Figure 1).

### 3.3 Prevalence of gastrointestinal parasite infection in cattle, sheep and goats

Out of the 57,500 fecal samples from individual ruminants analyzed, 30,147 animals were tested positive for one or more GIPs, with a pooled prevalence of 59.1% (95% CI, 54.6–63.6; 95% PI, 14.7–95.6) across CSG (Figure 4).

**Figure 4:**
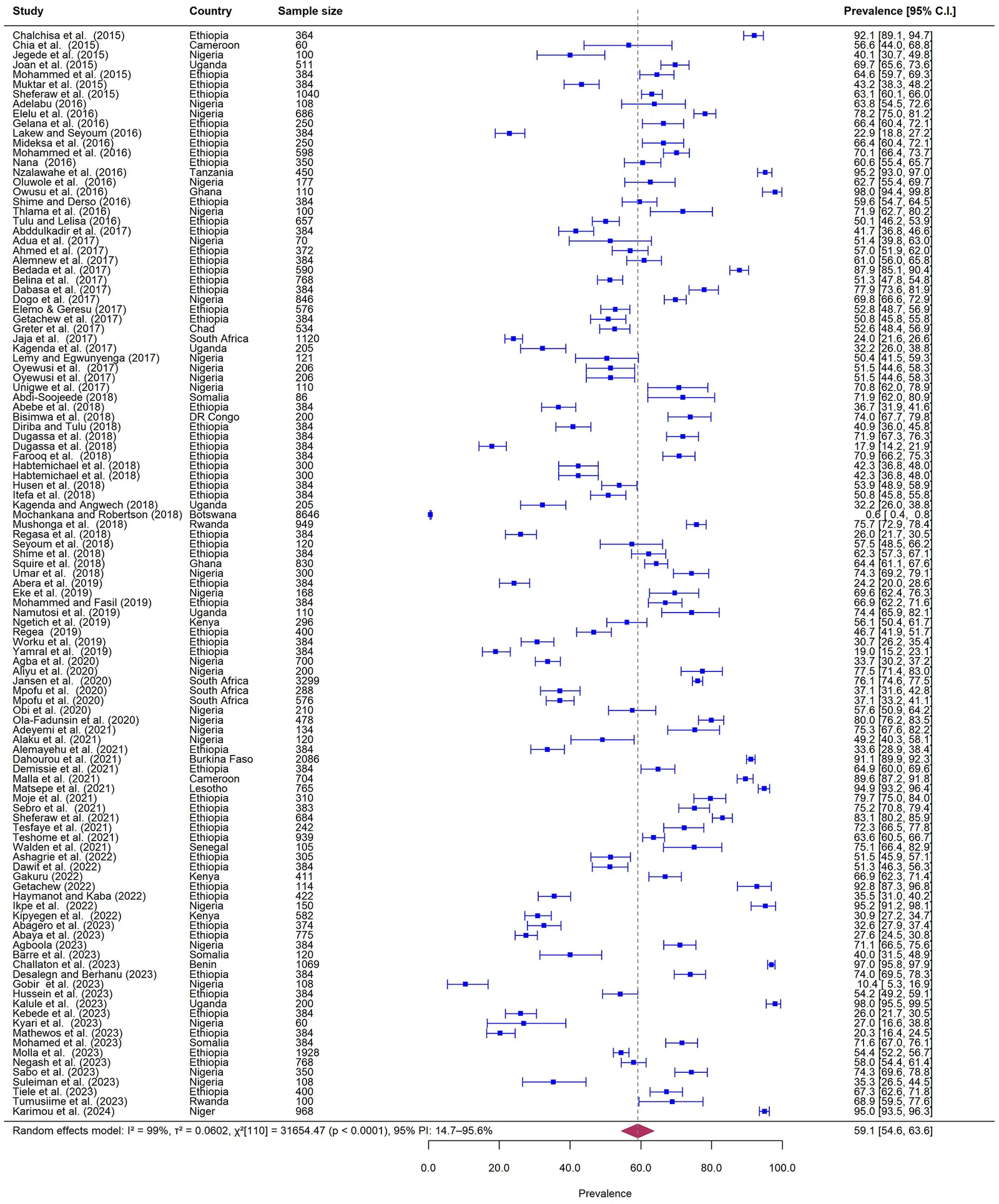
Forest plot of 111 studies included in a random-effects meta-analysis for pooled prevalence of infection with one or more gastrointestinal parasites in cattle, sheep, and goats combined across sub-Saharan Africa between 2015 and 2024. (Individual study estimates are ordered by publication year and then alphabetically by first author.)

At individual ruminant species level, the prevalence of GIP infection was 64.0% (95% CI, 58.7–69.2; 95% PI, 25.1–94.3) in sheep, 63.3% (95% CI, 56.3–70.1; 95% PI, 17.3–97.5) in goats, and 48.2% (95% CI, 41.2–55.1; 95% PI, 8.6–89.3) in cattle (Figures 5, 6).

**Figure 5:**
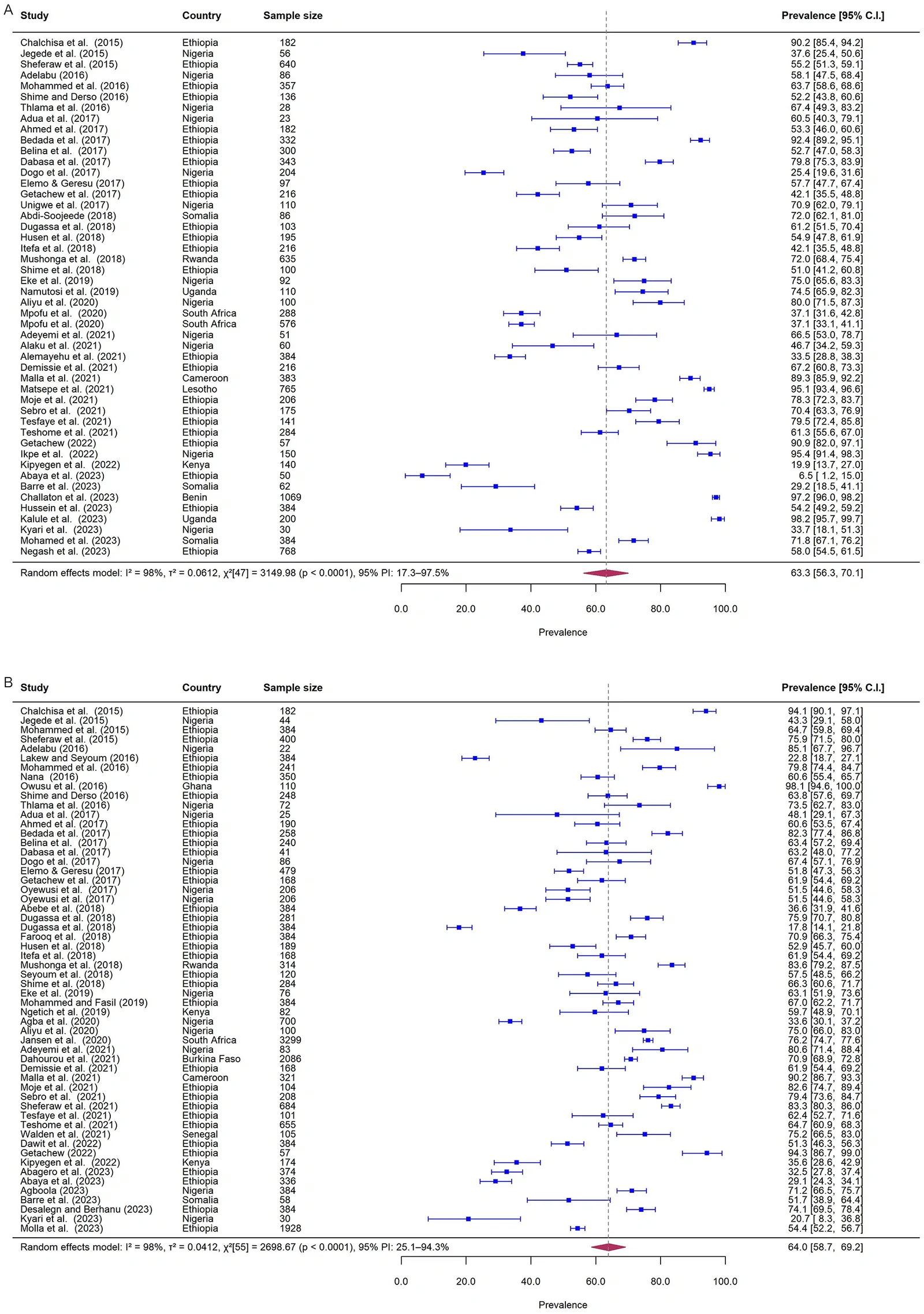
Forest plots of studies included in a random-effects meta-analysis for pooled prevalence of infection with one or more gastrointestinal parasites in goats **(A)** and sheep **(B)** across sub-Saharan Africa between 2015 and 2024. (Individual study estimates are ordered by publication year and then alphabetically by first author.)

**Figure 6:**
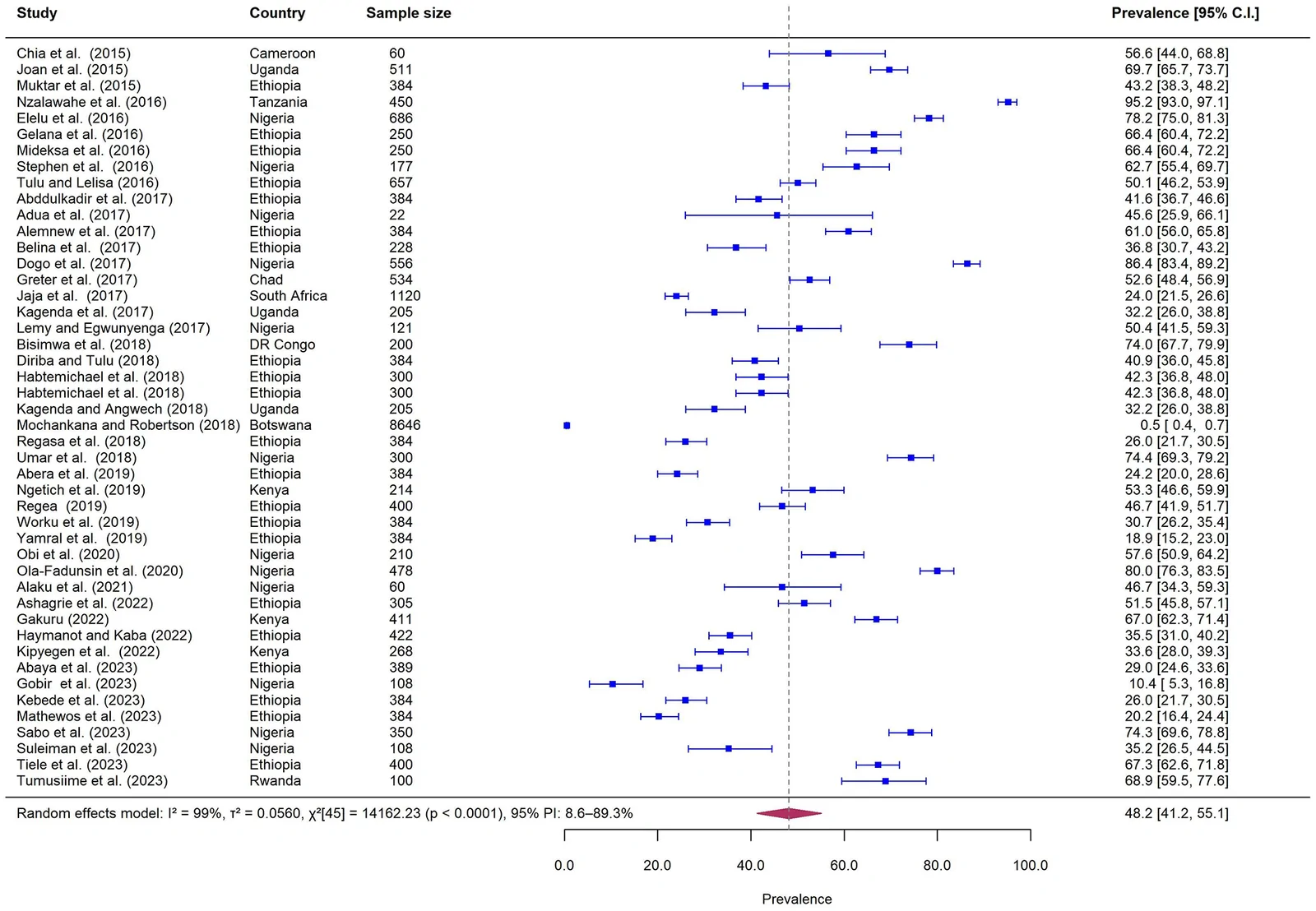
Forest plot of studies included in a random-effects meta-analysis for pooled prevalence of infection with one or more gastrointestinal parasites in cattle across sub-Saharan Africa between 2015 and 2024. (Individual study estimates are ordered by publication year and then alphabetically by first author.)

### 3.4 Subgroup analyses and Heterogeneity

Across CSG, the prevalence of GIP infection estimated by region ranged from 26.4% (95% CI, 25.7–27.2; 95% PI, 0.0–100) in Southern Africa to 74.0% (95% CI, 72.2–75.7; 95% PI, 35.1–96.2) in West Africa (Table 1).

**Table 1:** Subgroup meta-analysis of the pooled prevalence of fecal-detected gastrointestinal parasite taxa across cattle, sheep, and goats across sub-Saharan Africa, stratified by host species, geographical region, sample source, and fecal analytic method.

Species	Subgroup	Studies (*n*)	Sample size (*n*)	Prevalence (95% CI; 95% PI)	τ^2^	*I*^2^ (%)	Q (*p*-value)
All three species	Region						
	*Central Africa*	5	2447	74.0 (72.2–75.7; 35.1–96.2)	0.03	98.1	233 (< 0.001)
	*East Africa*	69	28991	55.0 (54.4–55.5; 16.4–91.6)	0.05	98.8	4839 (< 0.001)
	*Southern Africa*	6	14694	26.4 (25.7–27.2; 0.0–100.0)	0.19	99.9	12026 (< 0.001)
	*West Africa*	31	11368	74.9 (74.1–75.7; 20.1−98.6)	0.06	98.9	2546 (< 0.001)
	Sample source						
	*Abattoir*	21	5996	48.2 (45.9–48.4; 7.5–96.6)	0.09	98.9	1776 (< 0.001)
	*On-farm*	86	50480	52.6 (52.2–53.5; 15.7–95.8)	0.06	99.3	30802 (< 0.001)
	*Other*	4	1024	67.9 (64.9–70.7; 30.7–92.5)	0.03	95.9	86 (< 0.001)
	*Fecal analysis methods*						
	*Direct wet mount smear*	2	286	75.9 (70.5–80.7; NA)	NA	NA	NA
	*Flotation*	19	9051	69.2 (68.2–70.1; 18.7–99.7)	0.07	99.2	3050 (< 0.001)
	*Flotation & sedimentation*	79	44297	48.7 (48.2–49.1; 13.7–93.5)	0.06	99.2	25079 (< 0.001)
	*Immunodiagnostics (ELISA)*	1	775	61.4 (59.6–63.1; NA)	NA	NA	NA
	*Sedimentation*	10	3091	56.7 (39.5–73.1; 16.0–98.8)	0.06	98.6	752 (< 0.001)

Pooled estimates were obtained using a random-effects model; CI is the confidence interval; PI the prediction interval; τ^2^ the between-study variance; *I*^2^ the proportion of variability due to between-study heterogeneity; and Q the Cochran's heterogeneity statistic. Heterogeneity statistics are not estimable (i.e., NA) for subgroups with a single study.

By sample source, prevalence estimates for GIP infection were 48.2% (95% CI, 46.9–49.5; 95% PI, 7.5–96.6) for abattoir-based studies, 52.6% (95% CI, 52.2–53.1; 95% PI, 15.7–95.8) for on-farm studies, and 67.9% (95% CI, 64.9–70.7; 95% PI, 30.7–92.5) for other sources. The pooled prevalence also differed by fecal analysis method, with estimates being highest in studies that used direct wet mount smears 75.9% (95% CI, 70.5–80.7; 95% PI, not estimable) and flotation methods 69.2% (95% CI, 68.2–70.1; 95% PI, 18.7–99.7; Table 1). At individual ruminant species level, subgroup analyses by region revealed prevalence estimates ranging from 3.4%−71.2% in cattle, 62.2%−89.0% in goats, and 59.7%−90.0% in sheep (Table 2).

**Table 2:** Subgroup meta-analysis of the pooled prevalence of fecal-detected gastrointestinal parasite taxa in cattle, sheep, and goats across sub-Saharan Africa, stratified by host species, geographical region, sample source, and fecal analytic method.

Species	Subgroup	Studies (*n*)	Sample size (*n*)	Prevalence (95% CI; 95% PI)	τ^2^	*I*^2^ (%)	Q (*p*-value)
Cattle	Region						
	*Central Africa*	3	794	58.3 (54.8–61.8; 34.8–84.8)	0.01	91.2	29 (< 0.001)
	*East Africa*	29	10105	45.7 (44.7–46.7; 11.4–82.4)	0.04	98.2	1711 (< 0.001)
	*Southern Africa*	2	9766	3.4 (3.1–3.8; NA)	NA	NA	NA
	*West Africa*	12	3176	71.2 (69.6–72.8; 14.3–96.1)	0.06	98.3	440 (< 0.001)
	Sample source						
	*Abattoir*	12	3544	43.2 (41.6–44.8; 7.7–87.1)	0.05	98.2	725 (< 0.001)
	*On-farm*	33	19819	29.1 (28.4–29.7; 8.0–89.7)	0.06	99.1	12209 (< 0.001)
	*Other*	1	478	79.9 (76.0–83.4; NA)	NA	NA	NA
	Fecal analysis methods						
	*Flotation*	5	2185	44.5 (42.4–46.6; 13.5–88.5)	0.04	98.2	432 (< 0.001)
	*Flotation & sedimentation*	33	19998	28.5 (27.8–29.1; 6.6–88.0)	0.06	99.1	11589 (< 0.001)
	*Immunodiagnostics (ELISA)*	1	389	29.0 (24.6–33.8; NA)	NA	NA	NA
	*Sedimentation*	7	1269	71.0 (68.4–73.5; 17.1–97.1)	0.05	97.2	321 (< 0.001)
Goats	Region						
	*Central Africa*	1	383	89.0 (85.5–92.0; NA)	NA	NA	NA
	*East Africa*	31	7681	62.2 (61.1–63.3; 17.9–96.2)	0.06	98.2	1148 (< 0.001)
	*Southern Africa*	3	1629	64.2 (61.8–66.5; 0.0–100.0)	0.16	99.7	758 (< 0.001)
	*West Africa*	13	2059	79.3 (77.5–81.0; 15.9–99.0)	0.07	97.0	771 (< 0.001)
	Sample source						
	*Abattoir*	9	957	76.8 (74.0–79.4; 21.1–100)	0.06	96.3	217 (< 0.001)
	*On-farm*	38	10744	65.4 (64.5–66.3; 15.6–96.8)	0.06	98.6	2865 (< 0.001)
	*Other*	1	51	66.7 (52.1–79.2; NA)	NA	NA	NA
	Fecal analysis methods						
	*Direct wet mount smears*	2	186	76.3 (69.6–82.3; NA)	NA	NA	NA
	*Flotation*	11	4319	74.5 (73.1–75.8; 20.4–99.2)	0.06	98.9	1257 (< 0.001)
	*Flotation & sedimentation*	32	6987	61.0 (59.9–62.2; 18.9–95.7)	0.05	97.8	1365 (< 0.001)
	*Immunodiagnostics (ELISA)*	1	50	6.0 (1.3–16.5; 1.4–15.2)	NA	NA	NA
	*Sedimentation*	2	210	81.4 (75.5–86.4; NA)	NA	NA	NA
Sheep	Region						
	*Central Africa*	1	321	90.0 (86.2–93.1; NA)	NA	NA	NA
	*East Africa*	38	12154	59.7 (58.8–60.6; 24.7–93.1)	0.04	98.0	1749 (< 0.001)
	*Southern Africa*	1	3299	76.0 (74.5–77.4; NA)	NA	NA	NA
	*West Africa*	16	4335	63.2 (61.8−64.6; 22.2–96.3)	0.05	97.6	518 (< 0.001)
	Sample source						
	*Abattoir*	8	1495	41.6 (39.1–44.2; 11.4–91.6)	0.07	96.7	148 (< 0.001)
	*On-farm*	45	18119	65.6 (64.9–66.3; 27.5–94.9)	0.04	98.4	2164 (< 0.001)
	*Other*	3	495	56.4 (51.9–60.8; 23.5–92.8)	0.03	94.8	27 (< 0.001)
	Fecal analysis methods						
	*Direct wet mount smears*	1	100	75.0 (65.3–83.1; NA)	NA	NA	NA
	*Flotation*	10	1579	73.2 (71.0–75.4; 22.7–99.5)	0.06	97.2	173 (< 0.001)
	*Flotation & sedimentation*	42	17312	64.6 (63.9–65.3; 27.1–93.0)	0.04	98.3	1995 (< 0.001)
	*Immunodiagnostics (ELISA)*	1	336	29.2 (24.4–34.3; NA)	NA	NA	NA
	*Sedimentation*	2	782	36.4 (36.1–39.9; NA)	NA	NA	NA

Pooled estimates were obtained using a random-effects model; CI is the confidence interval; PI the prediction interval; τ^2^ the between-study variance; *I*^2^ the proportion of variability due to between-study heterogeneity; and Q the Cochran's heterogeneity statistic. Heterogeneity statistics are not estimable (i.e., NA) for subgroups with a single study.

Across all subgroup analyses, Cochran's Q tests were statistically significant (*p* < 0.001), except where subgroups contained less than three studies.

Heterogeneity testing across CSG revealed that *I*^2^ was 99.2% with an estimated between-study variance τ^2^ of 0.06, and the *Q*-statistic was 31,654 (*p* < 0.0001). Heterogeneity estimates for individual species was *I*^2^ = 98.3% in sheep (τ^2^ = 0.04, *Q* = 2,699 [*p* < 0.0001]), *I*^2^ = 98.3% in goats (τ^2^ = 0.06, *Q* = 3,150 [*p* < 0.0001]), and *I*^2^ = 99.0% in cattle (τ^2^ = 0.06, *Q* = 14,162 [*p* < 0.0001]).

### 3.5 Risk of publication bias and sensitivity analyses

Across all CSG combined, five studies (i.e., (41–45)) were identified as influential based on the studentized residuals and Cook's distance (Supplementary Table 4). At species level, five, three, and two influential studies were detected in goats, cattle, and sheep (Supplementary Table 5). After excluding the studies flagged as influential, the sensitivity analyses revealed that the overall prevalence estimate for GIP infection was 58.9% (95% CI, 54.7–62.9) across CSG (Table 3). Similarly, after excluding influential studies at individual species level, pooled prevalence changed from 48.2% to 48.4% in cattle, from 63.3% to 62.5% in goats, and from 64.0% to 64.7% in sheep (Table 3), with fully overlapping 95% CIs.

**Table 3:** Sensitivity analyses assessing the robustness of pooled prevalence estimates of fecal-detected gastrointestinal parasite infection in cattle, goats, and sheep across sub-Saharan Africa following the exclusion of influential studies and studies at moderate or high risk of bias.

Species	Sensitivity analysis scenario	No. of studies	Prevalence (95% CI)	*I*^2^ (%)
All	All studies (baseline)	111	59.1 (54.6–63.6)	99.2
	Excluding influential studies	106	58.9 (54.8–62.9)	98.9
	Excluding high-risk bias studies	105	59.0 (54.3–63.7)	99.2
	Using low-risk bias studies only	62	55.8 (49.1–62.4)	99.4
Cattle	All studies (baseline)	46	48.2 (41.3–55.1)	99.0
	Excluding influential studies	45	48.4 (42.5–54.4)	98.1
	Excluding high-risk bias studies	44	46.5 (39.7–53.4)	99.0
	Using low-risk bias studies only	28	45.9 (36.8–55.1)	99.2
Sheep	All studies (baseline)	56	64.0 (58.6–69.1)	98.3
	Excluding influential studies	51	64.7 (60.3–69.0)	97.3
	Excluding high-risk bias studies	52	64.9 (59.4–70.2)	98.4
	Using low-risk bias studies only	27	64.3 (56.8–71.4)	98.5
Goats	All studies (baseline)	48	63.3 (56.2–69.9)	98.3
	Excluding influential studies	45	62.5 (56.2–68.5)	97.6
	Excluding high-risk bias studies	43	65.1 (57.8–72.0)	98.4
	Using low-risk bias studies only	22	60.6 (49.7–71.1)	99.0

Pooled estimates were obtained using a random-effects model; CI is the confidence interval; and *I*^2^ the proportion of variability due to between-study heterogeneity.

The risk-of-bias sensitivity analyses showed that across all CSG, the prevalence remained virtually unchanged after excluding high-risk studies (59.1% vs. 59.0%) and when restricting the analysis to low-risk studies only (58.8%). Similar patterns were observed for cattle, sheep, and goats, with overlapping 95% CIs across the respective analyses (Table 3).

## 4 Discussion

Exposure to GIPs can lead to important health problems in grazing ruminants especially in SSA where the warm weather favors parasite proliferation. While several studies have been conducted at community/city level in several African countries, we are unaware of any study that systematically summarized evidence on the scale of gastrointestinal parasitism in the region. Thus, the present study is the first SSA-wide systematic evidence map and meta-analysis of fecal-detected GIP infections in cattle, sheep, and goats from studies published between 2015 and 2024. The discussion therefore considers the scale and distribution of infection, what the evidence map reveals about surveillance capacity and evidence gaps, and the implications for animal health, food safety, research, and targeted parasite control.

### 4.1 Regional burden of GIP infections, host-species patterns and heterogeneity

The overall pooled prevalence 59.1% (Figure 4) indicates that GIP infection is commonly detected in the available published evidence from SSA and reinforces the importance of parasitism as a major animal health constraint in the region (22, 23, 46). However, this pooled estimate should not be interpreted as a precise prevalence applicable to every country, livestock population, or production system in SSA. The very broad 95% prediction interval (14.7–95.6%) and extreme heterogeneity among studies (*I*^2^ = 99.2%) suggest that the expected prevalence in individual comparable settings may range from low to very high. This variation is plausible in a region encompassing highly diverse agroecological zones, production systems, animal populations, parasite communities, access to veterinary services, and diagnostic approaches (2, 37). Thus, our regional estimate is most useful as a summary of the scale of the problem across the available evidence base, rather than as a substitute for locally representative surveillance.

The present meta-analysis also showed a consistent host-species pattern, with higher pooled prevalence estimated in sheep (64.0%; 95% CI: 58.7–69.2) and goats (63.3%; 95% CI: 56.3–70.1) than in cattle (48.2%; 95% CI: 41.2–55.1). The overlapping CI for sheep and goats suggests that their regional average infection occurrence was broadly similar, indicating similar burden amongst small ruminants. This pattern is consistent with previous parasite-focused syntheses and epidemiological literature indicating that small ruminants often experience greater GIPs exposure and disease risk than cattle (47). This is likely due to the interaction between their behavioral, immunological, and physiological characteristics that influence exposure and host-parasite dynamic (48). For example, sheep and goats (particularly under conditions of limited forage availability) commonly graze closer to the ground and consume shorter herbage, potentially increasing contact with infective third-stage larvae concentrated near the herbage–soil interface compared to cattle. More so, differences in host animal characteristics (e.g., physiology, acquired immunity, body size, and nutritional status), as well as environmental and management factors (e.g., climate and husbandry practices) may also contribute to lower average fecal detection in cattle (47–50). Nevertheless, the present synthesis cannot attribute the observed species differences to any single biological, management, or environmental mechanism because these factors were not reported across the included studies. As such, the present species-specific results should guide surveillance attention towards sheep and goats rather than be interpreted as evidence of an inherent or uniform susceptibility difference across all production systems in SSA.

The marked regional variation further indicates that the burden of fecal-detected GIP infections was not spatially uniform in the available evidence base. The pooled estimates were highest in West (74.9%; 11,368 animals) and Central (74.0%; five studies; 2,447 animals) Africa, intermediate in East Africa (55.0%; 69 studies; 28,991 animals), and lowest in Southern Africa (26.4%; six studies; 14,694 animals). Similarly pronounced regional gradients were also observed within individual species (Table 2). Wetter and more humid conditions in parts of West and Central Africa may favor development and survival of free-living parasite stages and increase pasture contamination (51, 52). These environmental conditions may lead to higher pasture contamination and infection burdens in Central and West Africa than in drier Southern African systems. Moreover, regional differences in veterinary service coverage and anthelmintic use may reinforce these climatic effects (53). For example, irregular deworming and limited access to veterinary inputs in many smallholder systems in East and West Africa are associated with sustained high prevalence, whereas more structured parasite control programmes in some Southern African commercial systems likely contribute to lower infection burdens (53, 54). However, these explanations remain plausible hypotheses rather than causal inferences from the present dataset. The “region” subgroup variable is also confounded by differences in production systems, host-species composition, diagnostic methods, seasonality, and uneven country representation. In particular, estimates for some region-by-species combinations were based on one or only a few studies (Table 2), and should not be compared directly with estimates supported by dozens of studies. However, the pooled prevalence estimates for cattle in Southern Africa as well as goats and sheep in Central Africa should be interpreted cautiously. The estimate from Southern Africa was based on only two studies, albeit with a large combined sample size (9,766 animals), and it may therefore be strongly shaped by the characteristics of the sampled populations or production systems. It should not be read as evidence that gastrointestinal parasitism is unimportant in cattle throughout Southern Africa, nor as confirmation of successful regional parasite control. Instead, it highlights the need for representative studies spanning smallholder, pastoral, peri-urban, and commercial systems, as well as contrasting climatic zones and seasons. Similarly, the very high goat and sheep estimates reported for Central Africa (89.0% and 90.0%, respectively) each derive from a single study and are better interpreted as signals warranting further investigation than as stable regional benchmarks.

Furthermore, the pattern by sampling source underscores the relevance of GIP infection at both production and market/consumer levels. Pooled prevalence was 52.6% in on-farm studies (86 studies; 50,480 animals) and 48.2% in abattoir-based studies (21 studies; 5,996 animals), although both estimates had broad prediction intervals. The on-farm data suggest that fecal-detected parasitism is widespread in the CSG from which meat, milk, and manure are derived. The abattoir data indicate that infected animals commonly enter slaughter and therefore justify closer attention to abattoirs as complementary animal-health observation and surveillance points (55, 56). Yet abattoir CSG populations are selected animals for slaughter, which may differ from farm populations (e.g., in age, sex, and health status) (57). Consequently, abattoir and farm estimates should be treated as complementary evidence streams rather than interchangeable measures of population prevalence.

Heterogeneity was very high for CSG combined (*I*^2^ = 99.2%) and remained high at individual species level, geographic regions, sample sources, and fecal analysis method subgroups (Tables 1, 2). The broad 95% prediction intervals (i.e., 17.7–95.6% for CSG) further indicate that the underlying prevalence expected in a new comparable study setting could range from relatively low to very high. Such very high heterogeneity (i.e., between-study variability) has been repeatedly reported in geographically broad meta-analyses of ruminant parasitism. For example, Nambuya et al. (22) reported *I*^2^ > 99% in a meta-analysis of animal fascioliasis in Eastern and Southern Africa while Taghipour et al. (58) reported *I*^2^ > 98% in their study on *Giardia duodenalis* infect in cattle globally. In the present study, the persistence of high heterogeneity after stratification suggests that the broad categories of host species, region, sample source, and fecal diagnostic method did not adequately capture the more local determinants of infection. The observed variation likely reflects both genuine epidemiological differences among host populations and settings, and methodological or measurement-related differences among surveys (59). These may include differences in sampling frames, host age and physiological status, production system, management practices, season and agroecological conditions, diagnostic protocol and analytical performance, parasite-identification level, and the timing and handling of fecal samples. However, the independent contribution of most of these factors could not be quantified because reporting in the primary studies was either inconsistent or unavailable (e.g., weather data).

### 4.2 Evidence map of parasite taxa and surveillance gaps in SSA

Most included studies used flotation plus sedimentation (71.2%), followed by flotation alone (17.1%) and sedimentation alone (9.0%). These methods are practical and widely applicable in field-based surveillance, but routine coprological examination often identifies eggs or oocysts only to broad taxonomic levels for several important parasite groups, particularly strongylid nematodes (59). Consequently, genus-level identification dominated the available evidence, whereas species-specific information was comparatively sparse. Across CSG, *Strongyloides* (137 records) and *Fasciola* (79 records) were the most frequently reported genera, while *Trichuris, Eimeria*, and *Moniezia* were also commonly detected. Among the smaller subset of records reporting parasites at species level, *H. contortus* (18 records) and *F. gigantica* (15 records) were the most frequently identified. These findings are consistent with previous national and regional reviews which suggest that gastrointestinal parasitism in CSG across SSA is dominated by gastrointestinal nematodes, including strongylid/strongyle-type nematodes, and trematodes, particularly *Fasciola* spp., and Amphistomes (21–23). In cattle, *F. gigantica* and *S. bovis* were the most frequently reported species, while *Fasciola, Strongyloides*, and *Eimeria* were most frequently reported at genus level. In sheep and goats, *H. contortus* was the most frequently reported species, whereas *Strongyloides* and *Trichuris* were prominent at genus level. Together with previous parasite-specific evidence from SSA, our results support *Haemonchus*/strongyle-type nematodes as priority candidate targets for diagnosis and integrated parasite management in small ruminant systems. These findings also indicate that *Fasciola* spp. are a relevant target for surveillance in cattle and mixed ruminant systems, particularly in areas with suitable freshwater-snail habitats and ecological conditions for transmission (60). However, the predominance of genus-level identification in the present evidence base means that the true species composition of GIP communities in SSA is likely largely unknown, fundamentally limiting the capacity to implement targeted, species-specific control strategies.

Furthermore, the present study revealed that there are major geographical and surveillance gaps in terms of fecal-detected GIP infections in SSA. Although SSA encompasses 46 countries, the 111 eligible studies included in our evidence base were conducted in only 18 countries, with approximately 73% of them originating from Ethiopia and Nigeria (Figure 2). Thus, the apparent regional patterns observed in our results are based disproportionately on evidence from a small number of countries revealing major geographic gaps in the empirical evidence base on GIPs infection in CSG across SSA. For example, no eligible published studies were identified from several countries in the region, including Angola, Guinea, Mali, Mozambique, and Sudan. This absence should not be interpreted as evidence of absence of GIP infection or low parasite risk. Rather, it indicates that no accessible, English-language, fecal-based prevalence studies meeting the predefined eligibility criteria were identified by our study. This evidence gap may reflect limited or geographic uneven local surveillance, constraints in diagnostic capacity, limited international visibility of national research outputs in major bibliographic databases, or the limited accessibility of unpublished reports and theses in languages other than English (29, 61).

These geographic and taxonomic gaps have direct implications for veterinary-service planning and disease-burden assessment in SSA. First, countries with sparse evidence should be regarded as *evidence-priority settings* for representative baseline surveys, rather than being considered low-risk areas. Second, surveillance should progress beyond broad fecal detection (e.g., of strongyle-type eggs) toward diagnostic protocols capable of distinguishing priority parasites where this is epidemiologically justified. Third, future studies should report a minimum set of contextual variables (62), including: season, agroecological zone, host species details, production system, sample source, analytical detection threshold, and GIP species level identification inform targeted control strategies. Such reporting could be facilitated via adherence by authors to established reporting guidelines for observational studies, such as the “STrengthening the Reporting of OBservational Studies in Epidemiology (STROBE)” framework, to enhance transparency, reproducibility, and the analytical value of future studies.

### 4.3 One health, food safety, and socio-economic implications

First, the inclusion of both on-farm and abattoir-based studies in our evidence base broadens the scope of gastrointestinal parasitism from a livestock production constraint only to a multi-faceted food safety and public health concern. This is because abattoir-based studies reflect infected animals entering the food chain, whereas on-farm studies capture infection burdens within production systems. Across the 21 abattoir-based studies, comprising 5,996 animals, the pooled prevalence of infection with one or more fecal-detected GIP taxa was 48.2% (95% CI: 45.9–48.4%). This indicates that gastrointestinal parasitism is commonly detected among sampled slaughter animals in SSA. This finding is epidemiologically relevant because abattoirs are recognized interfaces for zoonotic exposure, as slaughter and carcass processing involve direct contact with intestinal contents, blood, offal, and fecal material (56). *Fasciola* spp. were among the most frequently reported genera across host species, and *F. gigantica* was the most frequently reported species in cattle. Fasciolosis is relevant to both livestock production and public health because *Fasciola* spp. are zoonotic parasites whose transmission is shaped by livestock infection, freshwater-snail ecology, water and vegetation use, and food-handling practices (60, 63). *S. bovis*, which was also frequently identified at species level in cattle records, is relevant for veterinary surveillance due to its capacity to hybridize with human schistosome where transmission systems overlap, as documented in West and Central Africa (64, 65). In contrast, the zoonotic relevance of records reported only as strongyle-type nematodes, *Strongyloides*, or *Trichuris* cannot be inferred from conventional fecal morphology alone; species-level confirmation and local epidemiological context are required before animal detections are translated into public-health risk statements. These findings support integrated farm- and abattoir-based parasite surveillance. Farm-level monitoring can guide locally appropriate control before animals enter market channels, whereas abattoir recording, targeted sampling, and animal-origin traceability can provide complementary information on parasite occurrence and potential transmission hotspots (55, 56). Combined with slaughter hygiene, worker protection, safe offal disposal, and farmer-level GIPs control, integrated surveillance could improve animal health, food safety, and environmental hygiene along ruminant value chains.

Second, beyond biological impacts, gastrointestinal parasitism has significant socio-economic implications. Using scored impacts, Perry et al. (66) ranked GIP infection as a major poverty alleviation constraint for smallholder livestock keepers in SSA. This is because GIP infections depreciate animal condition and performance, thereby limiting CSG farmers' chances to tap the growing market opportunities for animal-source foods but also their contribution to livestock-based sustainable intensification in SSA (11). In this context, the present findings suggest that prioritizing control of Strongyle-type nematodes, and *Fasciola* spp. represents a pragmatic entry point for improving animal health, food security, and incomes. However, important research gaps remain. For example, there are no clear and recent quantitative estimates on the impact of Strongyle-type nematodes and *Fasciola* spp. on productive and reproductive performance of CSG in SSA. As such, the true economic benefit of reducing GIPs prevalence in the region remains unclear. In addition, ruminants are under increasing scrutiny as major emitters of enteric methane, yet only one study to date has quantified the effect of gastrointestinal parasitism on methane emissions in sheep (67). Given the close relation between animal health, feed use efficiency, and greenhouse gas emissions, further research is needed to assess whether improved GIPs control could represent a low-cost co-benefit strategy for enhancing CSG productivity while reducing their associated enteric methane emissions in SSA.

### 4.4 Robustness and limitations of the present study

The sensitivity analyses indicated that the pooled estimates were robust to the exclusion influential studies and to restriction to risk of bias category (Table 3). Removing influential studies produced only negligible changes in pooled prevalence estimates across the combined CSG dataset and each host species, indicating that the overall estimates were not disproportionately determined by a small number of extreme studies. Similarly, excluding studies at high risk of bias or restricting analyses to studies at low risk of bias yielded broadly comparable estimates. The modest downward shifts observed for the combined CSG, cattle, and goat analyses when only low-risk studies were retained suggest that methodological quality may contribute to some variation in estimated prevalence; however, the persistence of moderate-to-high pooled estimates and overlapping confidence intervals supports the conclusion that frequent fecal detection of one or more GIP taxa is not attributable solely to studies with greater risk of bias. Nevertheless, these sensitivity analyses did not resolve the substantial between-study heterogeneity, rather they strengthen confidence that the main findings are robust across the available evidence base.

Three limitations of the present study must guide the interpretation of our findings. First, the review focused on studies using fecal-based diagnostics, excluding evidence generated solely through post-mortem inspection, serology, or other non-coprological methods. The results therefore describe the occurrence of fecal-detected gastrointestinal parasitism and should not be interpreted as a complete estimate of infection or disease burden for all GIPs taxa. Second, the English-language eligibility criterion and reliance on accessible literature may have excluded relevant evidence from Francophone and Lusophone countries, local journals, government reports, and unpublished theses. Third, most primary studies provided limited and inconsistent information on sampling frames, animal age and physiological status, husbandry practices, season, ecological context, and diagnostic implementation. This restricted our ability to explain heterogeneity, assess external validity, or identify causal determinants of infection occurrence using our data.

## 5 Conclusion

The present systematic evidence map and meta-analysis provides the first SSA-wide synthesis of fecal-detected GIP infection in CSG. Across 111 studies from 18 countries and 57,500 animals, the pooled prevalence of infection with one or more GIP taxa was 59.1% (95% CI: 54.6–63.6%), with higher estimates in sheep and goats than in cattle and substantial variation among regions and study settings. Given the very broad prediction intervals and extreme heterogeneity, this estimate should be interpreted as a regional average across diverse settings rather than as a directly transferable prevalence estimate for individual countries or production systems. The evidence mapping identified *Strongyloides* and *Fasciola* as the most frequently reported genera, and *H. contortus* and *F. gigantica* as the most frequently reported species among studies reporting species-level identification. These patterns suggest that *Haemonchus* and/or strongyle-type nematodes in small ruminants and *Fasciola* spp. in cattle and mixed-ruminant systems are suitable priority targets for improved diagnosis, surveillance, and locally adapted integrated control. Major geographic, diagnostic, and reporting gaps remain, with evidence concentrated in only 18 SSA countries. By identifying where fecal-based prevalence evidence is geographically sparse, diagnostically limited, or insufficiently resolved at species level, this evidence map provides a foundation for the more representative surveillance and burden data needed to support GBADs assessments of livestock disease impacts in SSA. Future work should prioritize representative surveillance in underrepresented settings, standardized diagnostics with improved species-level confirmation, and studies linking parasitism to productivity, economic loss, food safety, and anthelmintic resistance.

## Data Availability

The original contributions presented in the study are included in the article/Supplementary material, further inquiries can be directed to the corresponding author.
