# Supplementary material for: Prevalence of fecal-detected gastrointestinal parasites in cattle, sheep, and goats across sub-Saharan Africa: a systematic evidence map and meta-analysis
Source: Front Vet Sci. 2026 Oct 15;13:1984505. doi: 10.3389/fvets.2026.1984505 (PMC13717445; doi:10.3389/fvets.2026.1984505)
Supplement: Supplementary file 1 [file Table_1.docx]

**Supplementary Material**

**Supplementary Table 1**. PRISMA 2020 Checklist

| **Section and Topic** | **Item #** | **Checklist item** | **Location where item is reported** |
| --- | --- | --- | --- |
| **TITLE** | | |  |
| Title | 1 | Identify the report as a systematic review. | Title page |
| **ABSTRACT** | | |  |
| Abstract | 2 | See the PRISMA 2020 for Abstracts checklist. | Abstract |
| **INTRODUCTION** | | |  |
| Rationale | 3 | Describe the rationale for the review in the context of existing knowledge. | Introduction |
| Objectives | 4 | Provide an explicit statement of the objective(s) or question(s) the review addresses. | Introduction |
| **METHODS** | | |  |
| Eligibility criteria | 5 | Specify the inclusion and exclusion criteria for the review and how studies were grouped for the syntheses. | Section 2.2 |
| Information sources | 6 | Specify all databases, registers, websites, organisations, reference lists and other sources searched or consulted to identify studies. Specify the date when each source was last searched or consulted. | Section 2.3 |
| Search strategy | 7 | Present the full search strategies for all databases, registers and websites, including any filters and limits used. | Section 2.2 and supplementary material page 4 |
| Selection process | 8 | Specify the methods used to decide whether a study met the inclusion criteria of the review, including how many reviewers screened each record and each report retrieved, whether they worked independently, and if applicable, details of automation tools used in the process. | Section 2.4 |
| Data collection process | 9 | Specify the methods used to collect data from reports, including how many reviewers collected data from each report, whether they worked independently, any processes for obtaining or confirming data from study investigators, and if applicable, details of automation tools used in the process. | Section 2.4 |
| Data items | 10a | List and define all outcomes for which data were sought. Specify whether all results that were compatible with each outcome domain in each study were sought (e.g. for all measures, time points, analyses), and if not, the methods used to decide which results to collect. | Section 2.2 |
|  | 10b | List and define all other variables for which data were sought (e.g. participant and intervention characteristics, funding sources). Describe any assumptions made about any missing or unclear information. | Sections 2.2 - 2.4 |
| Study risk of bias assessment | 11 | Specify the methods used to assess risk of bias in the included studies, including details of the tool(s) used, how many reviewers assessed each study and whether they worked independently, and if applicable, details of automation tools used in the process. | Section 2.4 |
| Effect measures | 12 | Specify for each outcome the effect measure(s) (e.g. risk ratio, mean difference) used in the synthesis or presentation of results. | Section 2.4 |
| Synthesis methods | 13a | Describe the processes used to decide which studies were eligible for each synthesis (e.g. tabulating the study intervention characteristics and comparing against the planned groups for each synthesis (item #5)). | Sections 2.3 - 2.4 |
|  | 13b | Describe any methods required to prepare the data for presentation or synthesis, such as handling of missing summary statistics, or data conversions. | Sections 2.4 - 2.5 |
|  | 13c | Describe any methods used to tabulate or visually display results of individual studies and syntheses. | Sections 2.4 - 2.5 |
|  | 13d | Describe any methods used to synthesize results and provide a rationale for the choice(s). If meta-analysis was performed, describe the model(s), method(s) to identify the presence and extent of statistical heterogeneity, and software package(s) used. | Section 2.5 |
|  | 13e | Describe any methods used to explore possible causes of heterogeneity among study results (e.g. subgroup analysis, meta-regression). | Section 2.5 |
|  | 13f | Describe any sensitivity analyses conducted to assess robustness of the synthesized results. | Section 2.5 |
| Reporting bias assessment | 14 | Describe any methods used to assess risk of bias due to missing results in a synthesis (arising from reporting biases). | Section 2.5 |
| Certainty assessment | 15 | Describe any methods used to assess certainty (or confidence) in the body of evidence for an outcome. | Section 2.5 |
| **RESULTS** | | |  |
| Study selection | 16a | Describe the results of the search and selection process, from the number of records identified in the search to the number of studies included in the review, ideally using a flow diagram. | Section 3.1; Figure 1 |
|  | 16b | Cite studies that might appear to meet the inclusion criteria, but which were excluded, and explain why they were excluded. | Section 3.1; Figure 1 |
| Study characteristics | 17 | Cite each included study and present its characteristics. | Supplementary Table 3 |
| Risk of bias in studies | 18 | Present assessments of risk of bias for each included study. | Section 3.5, Table 2 - 3; Supplementary Table. 4 - 5 |
| Results of individual studies | 19 | For all outcomes, present, for each study: (a) summary statistics for each group (where appropriate) and (b) an effect estimate and its precision (e.g. confidence/credible interval), ideally using structured tables or plots. | Sections 3.3 - 3.4, Figures 4 - 6 |
| Results of syntheses | 20a | For each synthesis, briefly summarise the characteristics and risk of bias among contributing studies. | Section 3.5 |
|  | 20b | Present results of all statistical syntheses conducted. If meta-analysis was done, present for each the summary estimate and its precision (e.g. confidence/credible interval) and measures of statistical heterogeneity. If comparing groups, describe the direction of the effect. | Section 3.4 |
|  | 20c | Present results of all investigations of possible causes of heterogeneity among study results. | Section 3.4 |
|  | 20d | Present results of all sensitivity analyses conducted to assess the robustness of the synthesized results. | Section 3.5 |
| Reporting biases | 21 | Present assessments of risk of bias due to missing results (arising from reporting biases) for each synthesis assessed. | Section 3.5 |
| Certainty of evidence | 22 | Present assessments of certainty (or confidence) in the body of evidence for each outcome assessed. | Section 3.5 |
| **DISCUSSION** | | |  |
| Discussion | 23a | Provide a general interpretation of the results in the context of other evidence. | Sections 4.1 – 4.3 |
|  | 23b | Discuss any limitations of the evidence included in the review. | Sections 4.4 |
|  | 23c | Discuss any limitations of the review processes used. | Sections 4.4 |
|  | 23d | Discuss implications of the results for practice, policy, and future research. | Sections 4.1 – 4.3 |
| **OTHER INFORMATION** | | |  |
| Registration and protocol | 24a | Provide registration information for the review, including register name and registration number, or state that the review was not registered. | Not registered |
|  | 24b | Indicate where the review protocol can be accessed, or state that a protocol was not prepared. | Not prepared |
|  | 24c | Describe and explain any amendments to information provided at registration or in the protocol. | Not applicable |
| Support | 25 | Describe sources of financial or non-financial support for the review, and the role of the funders or sponsors in the review. | Statements |
| Competing interests | 26 | Declare any competing interests of review authors. | Statements |
| Availability of data, code and other materials | 27 | Report which of the following are publicly available and where they can be found: template data collection forms; data extracted from included studies; data used for all analyses; analytic code; any other materials used in the review. | Statements |

**Search query used to identify relevant studies to be included for the meta-analysis of the prevalence of gastrointestinal parasites in cattle, sheep, and goats across sub-Saharan Africa between 2015 – 2024.**

*Web of Science Search Query*

TS= (“Prevalence” OR “ubiquity” OR “frequency” OR “occurrence” OR “incidence”) AND TS=(“ gastrointestinal parasit*” OR “nematodes” OR “cestodes” OR “trematodes” OR “protozoa” OR “trichuris” OR “Eimeria” OR “fasciola” OR “gastrointestinal helminth*” OR “strongyl*”) AND TS =(“ruminant*” OR “cattle” OR “sheep” OR “goats”) AND CU= (Angola OR Benin OR Botswana OR Burkina Faso OR Burundi OR Cape Verde OR Cameroon OR Central African Republic OR Chad OR Comoros OR Congo OR Djibouti OR Equatorial Guinea OR Eritrea OR Eswatini OR Ethiopia OR Gabon OR Gambia OR Ghana OR Guinea OR Guinea-Bissau OR Ivory Coast OR Kenya OR Lesotho OR Liberia OR Madagascar OR Malawi OR Mali OR Mauritania OR Mauritius OR Mozambique OR Namibia OR Niger OR Nigeria OR Rwanda OR São Tomé and Príncipe OR Senegal OR Seychelles OR Sierra Leone OR Somalia OR South Africa OR South Sudan OR Sudan OR Tanzania OR Togo OR Uganda OR Zambia OR Zimbabwe) AND PY=(2013-2023)

*Google Scholar Search Query*

Prevalence of "gastrointestinal parasite*" in cattle, sheep and goats across sub-Saharan Africa “ubiquity” OR “frequency” OR “occurrence” OR “incidence” OR “ OR gastrointestinal OR parasit*” OR “nematodes” OR “cestodes” OR “trematodes” OR “protozoa” OR “trichuris” OR “Eimeria” OR “fasciola” OR “gastrointestinal OR helminth*” OR “strongyl*” OR “ruminant*” OR Angola OR Benin OR Botswana OR Burkina OR Faso OR Burundi OR Cape OR Verde OR Cameroon OR Central OR African OR Republic OR Chad OR Comoros OR Congo OR Djibouti OR Equatorial OR Guinea OR Eritrea OR Eswatini OR Ethiopia OR Gabon OR Gambia OR Ghana OR Guinea OR Guinea-Bissau OR Iv OR y OR Coast OR Kenya OR Lesotho OR Liberia OR Madagascar OR Malawi OR Mali OR Mauritania OR Mauritius OR Mozambique OR Namibia OR Niger OR Nigeria OR Rwanda OR São OR Tomé OR and OR Príncipe OR Senegal OR Seychelles OR Sierra OR Leone OR Somalia OR South OR Africa OR South OR Sudan OR Sudan OR Tanzania OR Togo OR Uganda OR Zambia OR Zimbabwe -review -"systematic review" -"meta-analyses"

**Supplementary Table 2.** *Item-level responses and overall risk-of-bias ratings for studies included in the meta-analysis, assessed using the Joanna Briggs Institute checklist for prevalence studies.*

| **Study  ID** | **Study Reference** | **Publication Year** | **Q1** | **Q2** | **Q3** | **Q4** | **Q5** | **Q6** | **Q7** | **Q8** | **Yes count** | **Bias risk** |
| --- | --- | --- | --- | --- | --- | --- | --- | --- | --- | --- | --- | --- |
| 1 | Tiele et al. | 2023 | Yes | Yes | Yes | Yes | Yes | Yes | Yes | No | 8 | Low |
| 2 | Tulu and Lelisa | 2016 | Yes | Yes | Yes | Yes | Yes | Yes | Yes | Yes | 8 | Low |
| 3 | Owusu et al. | 2016 | Yes | Unclear | No | Yes | Yes | Yes | Yes | No | 5 | Moderate |
| 4 | Habtemichael et al. | 2018 | Yes | Yes | Yes | Yes | Yes | Yes | Yes | No | 7 | Low |
| 5 | Regea | 2019 | Yes | Yes | Yes | Yes | Yes | Yes | Yes | No | 7 | Low |
| 6 | Obi et al. | 2020 | Yes | Yes | No | Yes | Yes | Yes | Yes | Yes | 7 | Low |
| 7 | Bisimwa et al. | 2018 | Yes | Yes | No | Yes | Yes | Yes | Yes | Yes | 7 | Low |
| 8 | Mideksa et al. | 2016 | Yes | Yes | No | Yes | Yes | Yes | Yes | No | 6 | Moderate |
| 9 | Kagenda et al. | 2018 | Yes | Yes | No | Yes | Yes | Yes | Yes | No | 6 | Moderate |
| 10 | Dogo et al. | 2017 | Yes | Unclear | Yes | Unclear | Yes | Yes | Unclear | No | 4 | High |
| 11 | Umar et al. | 2018 | Yes | Unclear | Yes | Yes | Yes | Yes | Yes | No | 6 | Moderate |
| 12 | Mpofu et al. | 2020 | Yes | Yes | Yes | Yes | Yes | Yes | Yes | Unclear | 7 | Low |
| 13 | Chia et al. | 2015 | Yes | Yes | No | No | Yes | Yes | Yes | No | 5 | Moderate |
| 14 | Dabasa et al. | 2017 | Yes | Yes | Yes | Yes | Yes | Yes | Yes | No | 7 | Low |
| 15 | Habtemichael et al. | 2018 | Yes | Yes | Yes | Yes | Yes | Yes | Yes | No | 7 | Low |
| 16 | Muktar et al. | 2015 | Yes | Yes | Yes | Yes | Yes | Yes | Yes | No | 8 | Low |
| 17 | Lemy and Egwunyenga | 2017 | Yes | No | No | Yes | Yes | Yes | Yes | No | 5 | Moderate |
| 18 | Aliyu et al. | 2020 | Yes | No | Yes | Yes | Yes | Yes | Yes | No | 6 | Moderate |
| 19 | Eke et al. | 2019 | Yes | No | No | Yes | Yes | No | Yes | No | 4 | High |
| 20 | Shime and Derso | 2016 | Yes | Yes | Yes | Yes | Yes | Yes | Yes | No | 7 | Low |
| 21 | Hussein et al. | 2023 | Yes | Yes | Yes | Yes | Yes | Yes | Yes | No | 7 | Low |
| 22 | Sebro et al. | 2022 | Yes | Unclear | Yes | Yes | Yes | Yes | Yes | No | 6 | Moderate |
| 23 | Abdi-Soojeede | 2018 | Yes | Unclear | No | Yes | Yes | No | Yes | No | 4 | High |
| 24 | Mushonga et al. | 2018 | Yes | Yes | Yes | Yes | Yes | Yes | Yes | No | 7 | Low |
| 25 | Jaja et al. | 2017 | Yes | Yes | Yes | Yes | Yes | Yes | Yes | No | 7 | Low |
| 26 | Matsepe et al. | 2021 | Yes | Yes | Yes | Yes | Yes | Yes | Yes | No | 8 | Low |
| 27 | Walden et al. | 2021 | Yes | No | No | Yes | Yes | Yes | Yes | Unclear | 5 | Moderate |
| 28 | Namutosi et al. | 2019 | Yes | No | No | Yes | Yes | Yes | Yes | Yes | 6 | Moderate |
| 29 | Mohamed et al. | 2023 | Yes | Unclear | Yes | Yes | Yes | Yes | Yes | Unclear | 6 | Moderate |
| 30 | Challaton et al. | 2023 | Yes | Yes | Yes | Yes | Yes | Yes | Yes | Unclear | 7 | Low |
| 31 | Joan et al. | 2015 | Yes | Yes | Yes | Yes | Yes | Yes | Yes | No | 7 | Low |
| 32 | Mohammed et al. | 2015 | Yes | Yes | Yes | Yes | Yes | Yes | Yes | Unclear | 7 | Low |
| 33 | Oyewusi et al. | 2017 | Yes | Yes | No | Yes | Yes | Yes | Yes | No | 6 | Moderate |
| 34 | Molla et al. | 2023 | Yes | Yes | Yes | Yes | Yes | Yes | Yes | Yes | 8 | Low |
| 35 | Jegede et al. | 2015 | Yes | Yes | No | Yes | Yes | Yes | Yes | No | 6 | Moderate |
| 36 | Moje et al. | 2021 | Yes | Yes | Yes | Yes | Yes | Yes | Yes | No | 7 | Low |
| 37 | Adua et al. | 2017 | Yes | Yes | No | Yes | Yes | Yes | Yes | No | 6 | Moderate |
| 38 | Kyari et al. | 2023 | Yes | Unclear | No | No | Yes | Yes | Yes | No | 4 | High |
| 39 | Abagero et al. | 2023 | Yes | Yes | Yes | Yes | Yes | Yes | Yes | Yes | 8 | Low |
| 40 | Abaya et al. | 2023 | Yes | Yes | Yes | Yes | Yes | Yes | Yes | No | 7 | Low |
| 41 | Abddulkadir et al. | 2017 | Yes | Yes | Yes | Yes | Yes | Yes | Yes | No | 7 | Low |
| 42 | Abebe et al. | 2018 | Yes | Yes | Yes | Yes | Yes | Yes | Yes | No | 7 | Low |
| 43 | Abera et al. | 2019 | Yes | Yes | Yes | Yes | Yes | Yes | Yes | No | 7 | Low |
| 44 | Adeyemi et al. | 2021 | Yes | Yes | No | Yes | Yes | Yes | Yes | No | 6 | Moderate |
| 45 | Agba et al. | 2020 | Yes | Unclear | Yes | Yes | Yes | Yes | Yes | No | 6 | Moderate |
| 46 | Ahmed et al. | 2017 | Yes | Unclear | No | Yes | Yes | Yes | Yes | No | 6 | Moderate |
| 47 | Alaku et al. | 2020 | Yes | Unclear | Yes | Yes | Yes | Yes | Yes | No | 6 | Moderate |
| 48 | Alemayehu et al. | 2021 | Yes | Yes | No | Yes | Yes | Yes | Yes | No | 6 | Moderate |
| 49 | Alemnew et al. | 2021 | Unclear | Yes | Yes | Yes | Yes | Yes | Yes | No | 7 | Low |
| 50 | Ashagrie et al. | 2017 | Yes | Yes | Yes | Yes | Yes | Yes | Yes | No | 7 | Low |
| 51 | Barre et al. | 2022 | Yes | Yes | Yes | Yes | Yes | Yes | Yes | No | 8 | Low |
| 52 | Belina et al. | 2023 | Yes | No | Yes | Unclear | Yes | Unclear | No | No | 2 | High |
| 53 | Dawit et al. | 2017 | Yes | Yes | Yes | Yes | Yes | Yes | Yes | No | 7 | Low |
| 54 | Demissie et al. | 2022 | Yes | Yes | Yes | Yes | Yes | Yes | Yes | No | 8 | Low |
| 55 | Desalegn and Berhanu | 2021 | Yes | Yes | Yes | Yes | Unclear | Yes | Unclear | No | 5 | Moderate |
| 56 | Sheferaw et al. | 2023 | Yes | Yes | Yes | Yes | Yes | Yes | Yes | No | 8 | Low |
| 57 | Diriba and Tulu | 2015 | Yes | Yes | Yes | Yes | Yes | Yes | Yes | No | 8 | Low |
| 58 | Dugassa et al. | 2018 | Yes | Yes | Yes | Yes | Yes | Yes | Yes | Yes | 8 | Low |
| 59 | Dugassa et al. | 2018 | Yes | Unclear | Yes | Yes | Yes | Yes | Yes | Unclear | 5 | Moderate |
| 60 | Getachew | 2018 | Yes | Unclear | Yes | Yes | Yes | Yes | Yes | Unclear | 5 | Moderate |
| 61 | Gobir et al. | 2022 | Yes | Yes | Yes | Yes | Yes | Yes | Yes | No | 7 | Low |
| 62 | Greter et al. | 2023 | Yes | Unclear | No | Yes | Yes | Yes | Yes | No | 5 | Moderate |
| 63 | Haymanot and Kaba | 2017 | Yes | Yes | Yes | Yes | Yes | Yes | Yes | Yes | 8 | Low |
| 64 | Ikpe et al. | 2022 | Yes | Yes | Yes | Yes | Yes | Yes | Yes | No | 7 | Low |
| 65 | Jansen et al. | 2022 | Yes | Yes | No | Yes | Yes | Yes | Yes | No | 6 | Moderate |
| 66 | Kagenda and Angwech | 2018 | Yes | Yes | No | Yes | Yes | Yes | Yes | No | 6 | Moderate |
| 67 | Kebede et al. | 2023 | Yes | Yes | Yes | Yes | Yes | Yes | Yes | Yes | 8 | Low |
| 68 | Kipyegen et al. | 2022 | Yes | Unclear | Yes | Yes | Yes | Yes | Yes | No | 6 | Moderate |
| 69 | Lakew and Seyoum | 2016 | Yes | Yes | Yes | Yes | Yes | Yes | Yes | Yes | 8 | Low |
| 70 | Malla et al. | 2021 | Yes | Unclear | Yes | Yes | Yes | Yes | Yes | No | 6 | Moderate |
| 71 | Mathewos et al. | 2023 | Yes | Yes | Yes | Yes | Yes | Yes | Yes | Unclear | 7 | Low |
| 72 | Gelana et al. | 2016 | Yes | Yes | No | Yes | Yes | Yes | Yes | No | 6 | Moderate |
| 73 | Mochankana and Robertson | 2018 | Yes | Yes | Yes | Yes | Yes | Yes | Yes | Yes | 8 | Low |
| 74 | Mohammed and Fasil | 2019 | Yes | Yes | Yes | Yes | Yes | Yes | Yes | No | 7 | Low |
| 75 | Mohammed et al. | 2016 | Yes | Yes | Yes | Yes | Yes | Yes | Yes | Yes | 8 | Low |
| 76 | Nana | 2016 | Yes | Yes | Yes | Yes | Yes | Yes | Yes | No | 7 | Low |
| 77 | Nzalawahe et al. | 2015 | Yes | Yes | Yes | Yes | Yes | Yes | Yes | No | 7 | Low |
| 78 | Oyewusi et al. | 2017 | Yes | Unclear | No | Yes | Yes | Yes | Yes | No | 5 | Moderate |
| 79 | Thlama et al. | 2016 | Yes | Yes | No | Yes | Yes | Yes | Yes | No | 6 | Moderate |
| 80 | Regasa et al. | 2018 | Yes | Yes | Yes | Yes | Yes | Yes | Yes | No | 7 | Low |
| 81 | Sabo et al. | 2023 | Yes | Unclear | Yes | Yes | Yes | Unclear | Yes | No | 4 | High |
| 82 | Seyoum et al. | 2018 | Yes | Yes | No | Yes | Yes | Yes | Yes | No | 6 | Moderate |
| 83 | Sheferaw et al. | 2021 | Yes | Yes | Yes | Yes | Yes | Yes | Yes | Yes | 8 | Low |
| 84 | Shime et al. | 2018 | Yes | Yes | Yes | Yes | Yes | Yes | Yes | No | 7 | Low |
| 85 | Stephen et al. | 2016 | Yes | Unclear | No | Yes | Yes | Yes | Yes | No | 5 | Moderate |
| 86 | Suleiman et al. | 2022 | Yes | Unclear | No | Yes | Yes | Yes | Yes | No | 6 | Moderate |
| 87 | Tesfaye et al. | 2021 | Yes | Yes | No | Yes | Yes | Yes | Yes | No | 6 | Moderate |
| 88 | Teshome et al. | 2021 | Yes | Unclear | Yes | Yes | Yes | Yes | Yes | No | 6 | Moderate |
| 89 | Tumusiime et al. | 2023 | Yes | Yes | Yes | Yes | Yes | Yes | Yes | No | 7 | Low |
| 90 | Unigwe et al. | 2017 | Yes | Yes | No | Yes | Yes | Unclear | Yes | No | 5 | Moderate |
| 91 | Worku et al. | 2019 | Yes | Yes | Yes | Yes | Yes | Yes | Yes | No | 7 | Low |
| 92 | Yamral et al. | 2016 | Yes | Yes | Yes | Yes | Yes | Yes | Yes | No | 7 | Low |
| 93 | Karimou et al. | 2024 | Yes | Yes | Yes | Yes | Yes | Yes | Yes | No | 7 | Low |
| 94 | Adelabu | 2016 | Yes | Unclear | No | Yes | Yes | Yes | Yes | No | 5 | Moderate |
| 95 | Agboola | 2023 | Yes | Unclear | Yes | Yes | Yes | Yes | Yes | No | 5 | Moderate |
| 96 | Bedada et al. | 2017 | Yes | No | Yes | Yes | Yes | Yes | Yes | No | 6 | Moderate |
| 97 | Chalchisa et al. | 2015 | Yes | Yes | Yes | Yes | Yes | Yes | Yes | No | 7 | Low |
| 98 | Dahourou et al. | 2021 | Yes | Yes | Yes | Yes | Yes | Yes | Yes | No | 7 | Low |
| 99 | Itefa et al. | 2018 | Yes | Yes | Yes | Yes | Yes | Yes | Yes | Unclear | 8 | Low |
| 100 | Elelu et al. | 2016 | Yes | Unclear | Yes | Yes | Yes | Yes | Yes | No | 5 | Moderate |
| 101 | Elemo & Geresu | 2017 | Yes | Yes | Yes | Yes | Yes | Yes | Yes | No | 7 | Low |
| 102 | Farooq et al. | 2018 | Yes | Yes | Yes | Yes | Yes | Yes | Yes | No | 7 | Low |
| 103 | Gakuru | 2022 | Yes | Yes | Yes | Yes | Yes | Yes | Yes | No | 7 | Low |
| 104 | Getachew et al. | 2017 | Yes | Yes | Yes | Yes | Yes | Yes | Yes | No | 7 | Low |
| 105 | Husen et al. | 2018 | Yes | Yes | Yes | Yes | Yes | Yes | Yes | Unclear | 7 | Low |
| 106 | Kalule et al. | 2023 | Yes | Unclear | No | Yes | Yes | Yes | Yes | Yes | 6 | Moderate |
| 107 | Mpofu et al. | 2020 | Yes | Yes | Yes | Yes | Yes | Yes | Yes | No | 7 | Low |
| 108 | Negash et al. | 2023 | Yes | Yes | Yes | Yes | Yes | Yes | Yes | Yes | 8 | Low |
| 109 | Ngetich et al. | 2019 | Yes | Yes | No | Yes | Yes | Yes | Yes | No | 6 | Moderate |
| 110 | Ola-Fadunsin et al. | 2020 | Yes | Yes | Yes | Yes | Yes | Yes | Yes | Yes | 8 | Low |
| 111 | Squire et al. | 2018 | Yes | Unclear | Yes | Yes | Yes | Yes | Yes | Yes | 6 | Moderate |

**Supplementary Table 3.** *Summary of studies on the prevalence of gastrointestinal parasites in cattle, sheep, and goats in sub-Saharan Africa*

| **Study  ID** | **Author(s)** | **Publication Year** | **Study Year*** | **Species** | **Study Country** | **Region** | **Sample Source** | **Sample Size** | **Infected** | **Prevalence (%)** |
| --- | --- | --- | --- | --- | --- | --- | --- | --- | --- | --- |
| 1 | Tiele et al. | 2023 | 2020 | Cattle | Ethiopia | East Africa | On-farm | 400 | 269 | 67.3 |
| 2 | Tulu and Lelisa | 2016 | 2008-2009 | Cattle | Ethiopia | East Africa | On-farm | 657 | 329 | 50.1 |
| 3 | Owusu et al. | 2016 | 2015 | Sheep | Ghana | West Africa | On-farm | 110 | 108 | 98.2 |
| 4 | Habtemichael et al. | 2018 | 2016-2017 | Cattle | Ethiopia | East Africa | On-farm | 300 | 127 | 42.3 |
| 5 | Regea | 2019 | 2018-2019 | Cattle | Ethiopia | East Africa | Abattoir | 400 | 187 | 46.8 |
| 6 | Obi et al. | 2020 | 2017 | Cattle | Nigeria | West Africa | Abattoir | 210 | 121 | 57.6 |
| 7 | Bisimwa et al. | 2018 | 2016 | Cattle | DR Congo | Central Africa | Abattoir | 200 | 148 | 74 |
| 8 | Mideksa et al. | 2016 | 2012-2014 | Cattle | Ethiopia | East Africa | On-farm | 250 | 166 | 66.4 |
| 9 | Kagenda et al. | 2017 | 2014-2015 | Cattle | Uganda | East Africa | On-farm | 205 | 66 | 32.2 |
| 10 | Dogo et al. | 2017 | 2009-2010 | Cattle, sheep,  & goats | Nigeria | West Africa | On-farm | 846 | 590 | 69.7 |
| 11 | Umar et al. | 2018 | 2016-2017 | Cattle | Nigeria | West Africa | Abattoir | 300 | 223 | 74.3 |
| 12 | Mpofu et al. | 2020 | NA | Goats | South Africa | Southern  Africa | On-farm | 576 | 74 | 12.8 |
| 13 | Chia et al. | 2015 | 2014 | Cattle | Cameroon | Central Africa | On-farm | 60 | 34 | 56.7 |
| 14 | Dabasa et al. | 2017 | 2016 | Sheep, goats | Ethiopia | East Africa | On-farm | 384 | 299 | 77.9 |
| 15 | Habtemichael et al. | 2018 | 2016-2017 | Cattle | Ethiopia | East Africa | On-farm | 300 | 127 | 42.3 |
| 16 | Muktar et al. | 2015 | 2011-2012 | Cattle | Ethiopia | East Africa | On-farm | 384 | 166 | 43.2 |
| 17 | Lemy and  Egwunyenga | 2017 | 2014-2015 | Cattle | Nigeria | West Africa | Abattoir | 121 | 61 | 50.4 |
| 18 | Aliyu et al. | 2020 | 2019 | Sheep, goats | Nigeria | West Africa | Abattoir | 200 | 155 | 77.5 |
| 19 | Eke et al. | 2019 | 2018 | Sheep, goats | Nigeria | West Africa | Abattoir | 168 | 117 | 69.6 |
| 20 | Shime and Derso | 2016 | 2013-2014 | Sheep, goats | Ethiopia | East Africa | On-farm | 384 | 229 | 59.6 |
| 21 | Hussein et al. | 2023 | 2020-2021 | Goats | Ethiopia | East Africa | On-farm | 384 | 208 | 54.2 |
| 22 | Sebro et al. | 2021 | NA | Sheep, goats | Ethiopia | East Africa | On-farm | 383 | 288 | 75.2 |
| 23 | Abdi-Soojeede | 2018 | 2018 | Goats | Somalia | East Africa | On-farm | 86 | 62 | 72.1 |
| 24 | Mushonga et al. | 2018 | 2014 | Goats | Rwanda | Central Africa | On-farm | 314 | 262 | 83.4 |
| 25 | Jaja et al. | 2017 | 2013-2014 | Cattle | South Africa | Southern Africa | Abattoir | 1120 | 420 | 37.5 |
| 26 | Matsepe et al. | 2021 | 2018-2019 | Goats | Lesotho | Southern Africa | On-farm | 765 | 725 | 94.8 |
| 27 | Walden et al. | 2021 | 2015 | Sheep | Senegal | West Africa | On-farm | 105 | 79 | 75.2 |
| 28 | Namutosi et al. | 2019 | NA | Goats | Uganda | East Africa | On-farm | 110 | 82 | 74.5 |
| 29 | Mohamed et al. | 2023 | 2020-2022 | Goats | Somalia | East Africa | On-farm | 384 | 275 | 71.6 |
| 30 | Challaton et al. | 2023 | 2019-2020 | Goats | Benin | West Africa | On-farm | 1069 | 1035 | 96.8 |
| 31 | Joan et al. | 2015 | 2014 | Cattle | Uganda | East Africa | Abattoir | 358 | 251 | 70.1 |
| 32 | Mohammed et al. | 2015 | 2014-2015 | Sheep | Ethiopia | East Africa | On-farm | 384 | 248 | 64.6 |
| 33 | Oyewusi et al. | 2017 | NA | Sheep | Nigeria | West Africa | Market | 206 | 106 | 51.5 |
| 34 | Molla et al. | 2023 | 2018 & 2021 | Sheep | Ethiopia | East Africa | On-farm | 1928 | 1049 | 54.4 |
| 35 | Jegede et al. | 2015 | 2014 | Sheep, goats | Nigeria | West Africa | On-farm | 100 | 40 | 40 |
| 36 | Moje et al. | 2021 | 2018-2019 | Sheep, goats | Ethiopia | East Africa | On-farm | 310 | 247 | 79.7 |
| 37 | Adua et al. | 2017 | 2016 | Cattle, sheep,  & goats | Nigeria | West Africa | Abattoir | 70 | 36 | 51.4 |
| 38 | Kyari et al. | 2023 | NA | Sheep, goats | Nigeria | West Africa | Abattoir | 60 | 16 | 26.7 |
| 39 | Abagero et al. | 2023 | 2023-2024 | Sheep | Ethiopia | East Africa | Abattoir | 374 | 122 | 32.6 |
| 40 | Abaya et al. | 2023 | 2020 | Cattle, sheep,  & goats | Ethiopia | East Africa | On-farm | 775 | 214 | 27.6 |
| 41 | Abddulkadir et al. | 2017 | 2014-2015 | Cattle | Ethiopia | East Africa | On-farm | 384 | 160 | 41.7 |
| 42 | Abebe et al. | 2018 | 2017-2018 | Sheep | Ethiopia | East Africa | On-farm | 384 | 141 | 36.7 |
| 43 | Abera et al. | 2019 | 2016-2017 | Cattle | Ethiopia | East Africa | On-farm | 384 | 93 | 24.2 |
| 44 | Adeyemi et al. | 2021 | 2018 | Sheep, goats | Nigeria | West Africa | Market | 134 | 101 | 75.4 |
| 45 | Agba et al. | 2020 | 2017-2018 | Sheep | Nigeria | West Africa | Abattoir | 700 | 236 | 33.7 |
| 46 | Ahmed et al. | 2017 | 2015-2016 | Sheep, goats | Ethiopia | East Africa | On-farm | 372 | 212 | 57 |
| 47 | Alaku et al. | 2021 | NA | Cattle, goats | Nigeria | West Africa | Abattoir | 120 | 58 | 48.3 |
| 48 | Alemayehu et al. | 2021 | 2021 | Goats | Ethiopia | East Africa | On-farm | 384 | 129 | 33.6 |
| 49 | Alemnew et al. | 2017 | 2014-2015 | Cattle | Ethiopia | East Africa | On-farm | 384 | 234 | 60.9 |
| 50 | Ashagrie et al. | 2022 | 2021-2022 | Cattle | Ethiopia | East Africa | On-farm | 305 | 157 | 51.5 |
| 51 | Barre et al. | 2023 | 2021-2022 | Sheep, goats | Somalia | East Africa | On-farm | 120 | 48 | 40 |
| 52 | Belina et al. | 2017 | 2015-2016 | Cattle, sheep,  & goats | Ethiopia | East Africa | On-farm | 768 | 394 | 51.3 |
| 53 | Dawit et al. | 2022 | 2018-2019 | Sheep | Ethiopia | East Africa | On-farm | 384 | 197 | 51.3 |
| 54 | Demissie et al. | 2021 | 2019-2020 | Sheep, goats | Ethiopia | East Africa | Abattoir | 384 | 249 | 64.8 |
| 55 | Desalegn and  Berhanu | 2023 | NA | Sheep | Ethiopia | East Africa | On-farm | 384 | 284 | 74 |
| 56 | Sheferaw et al. | 2015 | 2013-2014 | Sheep, goats | Ethiopia | East Africa | On-farm | 1040 | 656 | 63.1 |
| 57 | Diriba and Tulu | 2018 | 2015-2016 | Cattle | Ethiopia | East Africa | On-farm | 384 | 157 | 40.9 |
| 58 | Dugassa et al. | 2018 | 2016-2017 | Sheep, goats | Ethiopia | East Africa | On-farm | 384 | 276 | 71.9 |
| 59 | Dugassa et al. | 2018 | 2015-2016 | Sheep | Ethiopia | East Africa | On-farm | 384 | 69 | 18 |
| 60 | Getachew | 2022 | 2022 | Sheep, goats | Ethiopia | East Africa | On-farm | 114 | 106 | 93 |
| 61 | Gobir et al. | 2023 | NA | Cattle | Nigeria | West Africa | Abattoir | 108 | 11 | 10.2 |
| 62 | Greter et al. | 2017 | 2013-2014 | Cattle | Chad | Central Africa | On-farm | 534 | 281 | 52.6 |
| 63 | Haymanot and Kaba | 2022 | NA | Cattle | Ethiopia | East Africa | On-farm | 422 | 150 | 35.5 |
| 64 | Ikpe et al. | 2022 | 2013 | Goats | Nigeria | West Africa | Abattoir | 150 | 143 | 95.3 |
| 65 | Jansen et al. | 2020 | 2012-2015 | Sheep | South Africa | Southern Africa | On-farm | 3299 | 2506 | 76 |
| 66 | Kagenda and  Angwech | 2018 | 2014-2015 | Cattle | Uganda | East Africa | On-farm | 205 | 66 | 32.2 |
| 67 | Kebede et al. | 2023 | 2021-2022 | Cattle | Ethiopia | East Africa | On-farm | 384 | 100 | 26 |
| 68 | Kipyegen et al. | 2022 | NA | Cattle, sheep,  & goats | Kenya | East Africa | On-farm | 582 | 180 | 30.9 |
| 69 | Lakew and Seyoum | 2016 | 2014-2015 | Sheep | Ethiopia | East Africa | On-farm | 384 | 88 | 22.9 |
| 70 | Malla et al. | 2021 | NA | Sheep, goats | Cameroon | Central Africa | On-farm | 704 | 630 | 89.5 |
| 71 | Mathewos et al. | 2023 | 2020-2021 | Cattle | Ethiopia | East Africa | Abattoir | 384 | 78 | 20.3 |
| 72 | Gelana et al. | 2016 | 2012-2014 | Cattle | Ethiopia | East Africa | On-farm | 250 | 166 | 66.4 |
| 73 | Mochankana  and Robertson | 2018 | 2011-2013 | Cattle | Botswana | Southern  Africa | On-farm | 8646 | 64 | 0.7 |
| 74 | Mohammed  and Fasil | 2019 | 2015-2016 | Sheep | Ethiopia | East Africa | On-farm | 384 | 257 | 66.9 |
| 75 | Mohammed et al. | 2016 | 2010-2011 | Sheep, goats | Ethiopia | East Africa | On-farm | 598 | 419 | 70.1 |
| 76 | Nana | 2016 | 2014-2015 | Sheep | Ethiopia | East Africa | On-farm | 350 | 212 | 60.6 |
| 77 | Nzalawahe et al. | 2016 | 2012 | Cattle | Tanzania | East Africa | On-farm | 241 | 174 | 72.2 |
| 78 | Oyewusi et al. | 2017 | NA | Sheep | Nigeria | West Africa | Market | 206 | 110 | 53.4 |
| 79 | Thlama et al. | 2016 | NA | Sheep, goats | Nigeria | West Africa | On-farm | 100 | 72 | 72 |
| 80 | Regasa et al. | 2018 | 2016-2017 | Cattle | Ethiopia | East Africa | On-farm | 384 | 100 | 26 |
| 81 | Sabo et al. | 2023 | 2021 | Cattle | Nigeria | West Africa | On-farm | 350 | 260 | 74.3 |
| 82 | Seyoum et al. | 2018 | 2015-2016 | Sheep | Ethiopia | East Africa | On-farm | 120 | 69 | 57.5 |
| 83 | Sheferaw et al. | 2021 | 2014-2015 | Sheep | Ethiopia | East Africa | On-farm | 684 | 568 | 83 |
| 84 | Shime et al. | 2018 | 2016 | Sheep, goats | Ethiopia | East Africa | On-farm | 384 | 239 | 62.2 |
| 85 | Stephen et al. | 2016 | NA | Cattle | Nigeria | West Africa | On-farm | 177 | 111 | 62.7 |
| 86 | Suleiman et al. | 2023 | NA | Cattle | Nigeria | West Africa | Abattoir | 108 | 38 | 35.2 |
| 87 | Tesfaye et al. | 2021 | NA | Cattle | Ethiopia | East Africa | On-farm | 242 | 175 | 72.3 |
| 88 | Teshome et al. | 2021 | 2016-2017 | Sheep, goats | Ethiopia | East Africa | On-farm | 939 | 597 | 63.6 |
| 89 | Tumusiime et al. | 2023 | NA | Cattle | Rwanda | East Africa | On-farm | 100 | 69 | 69 |
| 90 | Unigwe et al. | 2017 |  | Goats | Nigeria | West Africa | On-farm | 110 | 78 | 70.9 |
| 91 | Worku et al. | 2019 | 2018-2019 | Cattle | Ethiopia | East Africa | On-farm | 384 | 118 | 30.7 |
| 92 | Yamral et al. | 2019 | 2014-2015 | Cattle | Ethiopia | East Africa | On-farm | 384 | 73 | 19 |
| 93 | Karimou et al. | 2024 | 2022 | Sheep, goats | Niger | West Africa | On-farm | 968 | 918 | 94.8 |
| 94 | Adelabu | 2016 | NA | Sheep, goats | Nigeria | West Africa | Abattoir | 108 | 96 | 88.9 |
| 95 | Agboola | 2023 | 2018 | Sheep | Nigeria | West Africa | On-farm | 384 | 273 | 71.1 |
| 96 | Bedada et al. | 2017 | 2014-2016 | Sheep, goats | Ethiopia | East Africa | On-farm | 590 | 518 | 87.8 |
| 97 | Chalchisa et al. | 2015 | 2014-2015 | Sheep, goats | Ethiopia | East Africa | On-farm | 364 | 335 | 92 |
| 98 | Dahourou et al. | 2021 | 2018-2019 | Sheep | Burkina  Faso | West Africa | On-farm | 2086 | 1898 | 91 |
| 99 | Itefa et al. | 2018 | 2016-2017 | Sheep, goats | Ethiopia | East Africa | On-farm | 384 | 195 | 50.8 |
| 100 | Elelu et al. | 2016 | 2013 | Cattle | Nigeria | West Africa | On-farm | 686 | 536 | 78.1 |
| 101 | Elemo & Geresu | 2017 | 2014-2015 | Sheep, goats | Ethiopia | East Africa | On-farm | 576 | 304 | 52.8 |
| 102 | Farooq et al. | 2018 | 2015-2016 | Sheep | Ethiopia | East Africa | On-farm | 384 | 272 | 70.8 |
| 103 | Gakuru | 2022 | 2020 | Cattle | Kenya | East Africa | On-farm | 411 | 275 | 66.9 |
| 104 | Getachew et al. | 2017 | 2013-2014 | Sheep, goats | Ethiopia | East Africa | On-farm | 384 | 195 | 50.8 |
| 105 | Husen et al. | 2018 | 2014-2015 | Sheep, goats | Ethiopia | East Africa | On-farm | 384 | 207 | 53.9 |
| 106 | Kalule et al. | 2023 | NA | Goats | Uganda | East Africa | Abattoir | 200 | 196 | 98 |
| 107 | Mpofu et al. | 2020 | NA | Goats | South Africa | Southern  Africa | On-farm | 288 | 106 | 36.8 |
| 108 | Negash et al. | 2023 | 2020-2021 | Goats | Ethiopia | East Africa | On-farm | 768 | 445 | 57.9 |
| 109 | Ngetich et al. | 2019 | 2016-2017 | Cattle, sheep | Kenya | East Africa | On-farm | 296 | 166 | 56.1 |
| 110 | Ola-Fadunsin et al. | 2020 | 2018-2019 | Cattle | Nigeria | West Africa | Abattoir & On-farm | 478 | 382 | 79.9 |
| 111 | Squire et al. | 2018 | 2014-2015 | Cattle, sheep,  & goats | Ghana | West Africa | On-farm | 830 | 534 | 64.3 |

*NA indicates that the year in which the study data were collected could not be determined, either directly or indirectly, from the available information.

**Full References of 111 Included studies**

1. Tiele, D., Sebro, E., H/Meskel, D., & Mathewos, M. (2023). Epidemiology of gastrointestinal parasites of cattle in and around Hosanna Town, Southern Ethiopia. Veterinary Medicine: Research and Reports, 14, 1–9. https://doi.org/10.2147/VMRR.S389787
2. Tulu, D., & Lelisa, K. (2016). A study on major gastro-intestinal helminths parasites of cattle in Tulo District, West Hararghe Zone, South-Eastern Ethiopia. Austin Journal of Veterinary Science and Animal Husbandry, 3(2), 3–6.
3. Owusu, M., Sekyere, J. O., & Adzitey, F. (2016). Prevalence and burden of gastrointestinal parasites of Djallonke sheep in Ayeduase, Kumasi, Ghana. Veterinary World, 9(4), 361–364. https://doi.org/10.14202/vetworld.2016.361-364
4. Habtemichael, Y. G., Dejene, M., & Eniyew, S. M. (2018). Prevalence of gastrointestinal helminth parasites and identification of major nematodes of cattle in and around Bishoftu, Oromia Region, Ethiopia. Journal of Veterinary Medicine and Animal Health, 10(7), 165–172. https://doi.org/10.5897/JVMAH2018.0690
5. Regea, G. (2019). Prevalence of major gastrointestinal tract parasite of cattle at Municipal Abattoir of Jimma Town, Oromia, South Western Ethiopia. Veterinary Medicine – Open Journal, 4(1), 36–44. https://doi.org/10.17140/VMOJ-4-134
6. Obi, C. F., Akata, M. C., & Ezubelu, O. J. (2020). Prevalence of gastrointestinal helminth parasites of trade cattle in Aguata and Orumba South Local Government Areas, Southeastern Nigeria. Journal of Parasitic Diseases, 44(3), 546–552. https://doi.org/10.1007/s12639-020-01227-3
7. Bisimwa, N. P., Lugano, R. M., Bwihangane, B. A., Wasso, S. D., Kinimi, E., Banswe, G., & Bajope, B. (2018). Prevalence of gastro-intestinal helminths in slaughtered cattle in Walungu Territory, South Kivu Province, Eastern Democratic Republic of Congo. Austin Journal of Veterinary Science & Animal Husbandry, 5(1), 1039.
8. Milkessa, G., Tesfaye, M., Tesfaye, M., Abera, H., Duguma, M., & Gudeta, T. (2016). Epidemiological study of gastro-intestinal helminthes parasites of calves in urban, peri-urban and rural smallholder dairy farms of East Wollega Zone, Western Ethiopia. Journal of Biology, Agriculture and Healthcare, 6(17), 29–34.
9. Kagenda, G. A., & Angwech, H. (2018). Cross-sectional prevalence of gastrointestinal helminth parasites in cattle in Lira District, Uganda. Tropical Animal Health and Production, 50, 1599–1604. https://doi.org/10.1007/s11250-018-1600-0
10. Dogo, G. I., Abraham, K. P., Gloria, P., Patrobas, M. G., Galadima, M., & Gosomji, I. J. (2017). Prevalence of gastrointestinal parasites and their impact in domestic animals in Vom, Nigeria. Saudi Journal of Medical & Pharmaceutical Sciences, 3(3B), 211–216. https://doi.org/10.36348/sjmps.2017.v03i03.016
11. Yuguda, A. U., Samaila, A. B., & Panda, S. M. (2018). Gastrointestinal helminths of slaughtered cattle in Bauchi Central Abattoir, Bauchi State, Nigeria. GSC Biological and Pharmaceutical Sciences, 4(2), 58–65. https://doi.org/10.30574/gscbps.2018.4.2.0036
12. Mpofu, T. J., Nephawe, K. A., & Mtileni, B. (2020). Prevalence of gastrointestinal parasites in communal goats from different agro-ecological zones of South Africa. Veterinary World, 13(1), 26–32. https://doi.org/10.14202/vetworld.2020.26-32
13. Chia, P. N., Ukaga, C. N., Yongabi, K. A., Nwoke, B. E. B., & Tih, P. M. (2015). Prevalence of Cryptosporidium parvum in dung collected from cattle in Bamenda, Northwest Region of Cameroon. Indian Journal of Medical Research and Pharmaceutical Sciences, 2(9), 1–15.
14. Dabasa, G., Shanko, T., Zewdei, W., Jilo, K., Gurmesa, G., & Abdela, N. (2017). Prevalence of small ruminant gastrointestinal parasites infections and associated risk factors in selected districts of Bale Zone, south-eastern Ethiopia. Journal of Parasitology and Vector Biology, 9(6), 81–88.
15. Habtemichael, Y. G., Dejene, M., & Eniyew, S. M. (2018). Prevalence of gastrointestinal helminth parasites and identification of major nematodes of cattle in and around Bishoftu, Oromia Region, Ethiopia. Journal of Veterinary Medicine and Animal Health, 10(7), 165–172. https://doi.org/10.5897/JVMAH2018.0690
16. Muktar, Y., Belina, D., Alemu, M., Shiferaw, S., & Belay, H. (2015). Prevalence of gastrointestinal nematode of cattle in selected kebeles of Dire Dawa districts, Eastern Ethiopia. Advances in Biological Research, 9(6), 418–423. https://doi.org/10.5829/idosi.abr.2015.9.6.96213
17. Lemy, E. E., & Egwunyenga, A. O. (2017). Prevalence of parasitic helminthes from feacal samples of cattle at various abattoirs in Abraka, Delta State, Nigeria. Journal of Animal Health and Behavioural Science, 1(3), 107.
18. Aliyu, A. A., Maikenti, J. I., Aimankhu, O. P., Ayuba, S. O., Ahmed, H. O., Haruna, A., & Idris, A. M. (2020). Helminth parasites of goats and sheep at slaughter house in Lafia, Nasarawa State, Nigeria. FUDMA Journal of Sciences, 4(2), 34–40. https://doi.org/10.33003/fjs-2020-0402-154
19. Eke, S. S., Omalu, I. C. J., Ochaguba, J. E., Urama, A. C., Hassan, S. C., Otuu, C. A., & Okafor, I. D. (2019). Prevalence of gastrointestinal parasites of sheep and goats slaughtered in Minna Modern Abattoir, Niger State, Nigeria. Journal of Animal Science and Veterinary Medicine, 4(2), Article DC4787863. https://doi.org/10.31248/JASVM2019.131
20. Shime, A. & Derso, D. (2017). Small ruminant GIT parasites in Enemay District, East Gojjam: Prevalence and risk factors. Online Journal of Animal and Feed Research, 7(3), 65–71.
21. Hussein, H. A., Abdi, S. M., Ahad, A. A., & Mohamed, A. (2023). Gastrointestinal nematodiasis of goats in Somali pastoral areas, Ethiopia. Parasite Epidemiology and Control, 23, e00324. https://doi.org/10.1016/j.parepi.2023.e00324
22. Sebro, E., Kebamo, M., & Abebe, A. (2022). Prevalence of gastrointestinal parasites of sheep and goats in An-Lemo, Hadiya Zone, Southern Ethiopia. Indian Journal of Science and Technology, 15(22), 1084–1090. https://doi.org/10.17485/IJST/v15i22.1384
23. Abdi-Soojeede, M. I. (2018). Common gastro-intestinal parasites of goats (Capra aegagrus hircus) from Mogadishu, Somalia. Open Journal of Veterinary Medicine, 8(12), 232-240
24. Mushonga, B., Niyonzima, R., Karugaba, J., Izimpaka, E., Muhindo, J., & Ntampaka, P. (2018). Prevalence of Haemonchus contortus infections in sheep and goats in Nyagatare District, Rwanda. Journal of Veterinary Medicine, 2018, Article ID 3602081. https://doi.org/10.1155/2018/3602081
25. Jaja, I. F., Mushonga, B., Green, E., & Muchenje, V. (2017). Seasonal prevalence, body condition score and risk factors associated with fasciolosis in cattle slaughtered at three abattoirs in South Africa. Veterinary Parasitology: Regional Studies and Reports, 8, 45–51. https://doi.org/10.1016/j.vprsr.2017.06.001
26. Matsepe, L. G., Molapo, S., Phalatsi, M., & Phororo, M. (2021). Prevalence and fecal egg load of gastrointestinal parasites of Angora goats in four agro-ecological zones in Lesotho. Veterinary World, 14(2), 339–346. https://doi.org/10.14202/vetworld.2021.339-346
27. Walden, H. D. S., & colleagues. (2021). Anemia and intestinal parasites in farmers and family members owned sheep in Diawara. One Health, 13, 100260. https://doi.org/10.1016/S2352-7714(21)00050-1
28. Namutosi, W., Higenyi, J., Kizito, E., & Omodo, M. (2019). Prevalence and risk factors of gastrointestinal parasite infection in goats in Sironko District, Eastern Uganda. Uganda Journal of Agricultural Sciences, 19(1), 1–14. https://doi.org/10.4314/ujas.v19i1.1
29. Sheikh Mohamed, M. O. S., Ibrahim, A. M., Ahmed, H. A., Yusuf, M. A. Y., Mio, J. B., Salah, O. M., Abdirahman, Z. M., & Hassan, Y. H. S. (2023). Assessment of the prevalence and associated risk factors of gastrointestinal parasite of goats in Afgooye District, Lower Shabelle, Somalia. Journal of Applied Veterinary Science and Technology, 4(2), 93–99. https://doi.org/10.20473/javest.V4.I2.2023.93-99
30. Challaton, K. P., Akouedegni, C. G., Boko, C. K., Alowanou, G. G., Houndonougbo, P. V., Kifouly, A. H., & Hounzangbé-Adoté, M. S. (2023). Evaluation of the gastrointestinal parasite burden of goats in traditional breeding in Benin. Journal of Animal Health and Production, 11(2), 144–154. https://doi.org/10.17582/journal.jahp/2023/11.2.144.154
31. Nambafu, J., Musisi, J. S., Mwambi, B., Kiguli, J., Orikiriza, P., Bazira, J., Itabangi, H., & Iramiot, J. S. (2015). Prevalence and economic impact of bovine fasciolosis at Kampala City Abattoir, Central Uganda. Microbiology Research Journal International, 7(3), 109–117. https://doi.org/10.9734/BMRJ/2015/15274
32. Mohammed, A., & Disassa, H. (2015). Prevalence of gastrointestinal nematodes of sheep in Gursum Woreda of Eastern Hararghe Zone, Oromia Regional State, Ethiopia. Researcher, 7(8), 45–54.
33. Oyewusi, I. K., Takeet, M. I., Olugbogi, E. I., Takeet, O. V. A., & Talabi, A. O. (2017). Prevalence of gastrointestinal parasites of rams brought into Abeokuta small ruminants markets in preparation for a festive season. Nigerian Journal of Animal Science, 19(1), 101-107.
34. Molla, W., Moliso, M. M., Gizaw, S., Nane, T., Arke, A., Ayele, F., & Knight-Jones, T. (2023). Ovine gastrointestinal parasite burden and the impact of strategic anthelmintic treatment in community-based breeding sites in Ethiopia. Frontiers in Veterinary Science, 10, 1094672. https://doi.org/10.3389/fvets.2023.1094672
35. Jegede, O. C., Adejoh, A. A., Obeta, S. S., & Olayemi, O. D. (2015). Gastrointestinal parasites of sheep and goats in Gwagwalada Area Council, Federal Capital Territory, Abuja, Nigeria: With a special reference to sex, breed and age. Alexandria Journal of Veterinary Sciences, 46(1), 170–176. https://doi.org/10.5455/ajvs.177135
36. Moje, N., Gurmesa, A., & Regassa, G. (2021). Gastro-intestinal tract nematodes of small ruminants: Prevalence and their identification in and around Alage, Southern Ethiopia. Animal and Veterinary Sciences, 9(3), 65–72. https://doi.org/10.11648/j.avs.20210903.14
37. Adua, M. M., Idahor, K. O., & Christopher, S. K. (2017). Assessment of roundworm species prevalence in cattle, sheep and goats slaughtered at Lafia Abattoir, Nasarawa State, Nigeria. Journal of Animal Science and Veterinary Medicine, 2(6). https://doi.org/10.31248/JASVM2017.075
38. Kyari, A. G., Adamu, A. H., Abubakar, A. Y., & Abdullahi, M. B. (2023). Prevalence of nematode (roundworm species) in slaughtered sheep and goats in Damaturu Modern Abattoir. FANE-FANE International Multidisciplinary Journal, 7(1), 43–48.
39. Abagero, F. Z., & colleagues. (2023). Prevalence, associated risk factors, and economic loss of ovine hemonchosis at Jimma Town Municipal Abattoir, Jimma Zone, Southwestern Ethiopia. Veterinary Medicine International, 2023, Article 9946518. https://doi.org/10.1155/2023/9946518
40. Abaya, S. W., Mereta, S. T., Tulu, F. D., Mekonnen, Z., Ayana, M., Girma, M., Vineer, H. R., Mor, S. M., Caminade, C., & Graham-Brown, J. (2023). Prevalence of human and animal fasciolosis in Butajira and Gilgel Gibe Health Demographic Surveillance System sites in Ethiopia. Tropical Medicine and Infectious Disease, 8(4), 208. https://doi.org/10.3390/tropicalmed8040208
41. Abdulkadir, O., Hamid, M., Alemayehu, A., & Tintagu, T. (2017). Study on the prevalence of GIT nematodes on bovine in and around Kombolcha and Dessie town, North Eastern, Ethiopia. Journal of Veterinary Science & Technology, 8(5), Article 477. https://doi.org/10.4172/2157-7579.1000477
42. Abebe, T., Yobsan, T., & Debela, A. (2018). Prevalence of major gastrointestinal nematode and degree of parasite infestation in sheep of Bako Agricultural Research Center community-based breeding program small holder farms at Horro District. Dairy and Veterinary Sciences Journal, 8(3), 555740. https://doi.org/10.19080/JDVS.2018.08.555740
43. Abera, A., & Gebrewahd, T. T. (2019). Prevalence and risk factors of ectoparasites in small ruminants in and around Haramaya University, Eastern Oromia Region, Ethiopia. Ethiopian Veterinary Journal, 23(1), 78–89.
44. Adeyemi, O. O., Idowu, E. T., Ikenweiwe, J. C., & Otubanjo, O. A. (2021). Status of Eimeria infections of sheep and goat sold at small ruminant markets in Lagos State, Southwest, Nigeria. Nigerian Journal of Parasitology, 42(1), 137–146. https://doi.org/10.4314/njpar.v42i1.19
45. Agba, A., Aguh, I., & Uweh, P. (2020). Abattoir survey of intestinal parasites among sheep in Katsina State, Nigeria. Nigerian Annals of Pure and Applied Sciences, 3(1), 97–102. https://doi.org/10.46912/napas.180
46. Ahmed, J., Duguma, A., Regassa, D., Belina, D., & Jilo, R. (2017). Gastrointestinal nematode parasites of small ruminants and anthelmintics efficacy test in sheep of Haramaya District, Eastern Ethiopia. Animal and Veterinary Sciences, 5(3), 39–44. https://doi.org/10.11648/j.avs.20170503.11
47. Alaku, A. I., Danladi, S., & Emmanuel, J. N. (2021). Assessment of gastrointestinal parasitic infection of cattle and goat slaughtered at Akwanga Abattoir, Nasarawa State, Nigeria. Asian Journal of Research in Animal and Veterinary Sciences, 4(4), 536–541. https://doi.org/10.9734/ajravs/2021/v4i4182
48. Alemayehu, A., Shiferaw, J., Feissa, A., & Tolossa, Y. H. (2021). Clinico-coproscopic study on major GIT helminthes of goats and their management approaches at Mojo Veterinary Clinic, Oromia Region, Ethiopia. International Journal of Advanced Research in Biological Sciences, 8(10), 64–70. https://doi.org/10.22192/ijarbs.2021.08.10.008
49. Alemnew, E., Delil, F., & Addis, H. (2017). Prevalence of bovine coccidiosis and ostertagiosis in and around Kombolcha district, South Wollo, Ethiopia. Academia Arena, 9(11), 16–25. https://doi.org/10.7537/marsaaj091117.03
50. Ashagrie, T., Zewde, D., Yemanebrhan, H., Yenew, M., & Yalew, B. (2022). Prevalence and species identification of calves eimeriosis in Sebeta Town, Oromia Special Zone, Ethiopia. Austin Journal of Veterinary Science & Animal Husbandry, 9(5), Article 1106. https://doi.org/10.29011/2577-9394.000106
51. Barre, A., Hirabe, A. M., Mohamed, A. A., Ibrahim, A. A., Mohamed, H. E., Adan, M. A., & Mohamed, N. A. (2023). Prevalence, public health and associated risk factors of coccidiosis in small ruminants at Deyniile sub-district in Mogadishu, Somalia. Journal of Veterinary Medicine and Animal Sciences, 6(1), 1–6.
52. Belina, D., Giri, A., Mengistu, S., & Eshetu, A. (2017). Gastrointestinal nematodes in ruminants: The parasite burden, associated risk factors and anthelmintic utilization practices in selected districts of East and Western Hararghe, Ethiopia. Journal of Veterinary Science & Technology, 8(2), 433. https://doi.org/10.4172/2157-7579.1000433
53. Dawit, I., Weldegebriel, M., Dejene, D., & Israel, I. (2022). Prevalence of gastrointestinal tract nematodes parasites in sheep in Hawasa Town, Southern Ethiopia. Biomedical Journal of Scientific & Technical Research, 41(5), 33046–33052. https://doi.org/10.26717/BJSTR.2022.41.006661
54. Demissie, T., Wakjira, B., Shiferaw, J., Feyisa, A., & Tolossa, Y. H. (2021). Pre-slaughter coproscopic and abattoir prevalence of GIT parasites of sheep and goats at Bishoftu ELFORA export abattoir, Oromia, Ethiopia. International Journal of Current Research in Biology and Medicine, 6(1), 12–17. https://doi.org/10.22192/ijcrbm.2021.06.01.002
55. Desalegn, C., & Berhanu, G. (2023). Assessment of the epidemiology of the gastrointestinal tract nematode parasites in sheep in Toke Kutaye, West Shoa Zone, Ethiopia. Veterinary Medicine: Research and Reports, 14, 177–183. https://doi.org/10.2147/VMRR.S427828
56. Sheferaw, D., Guteta, M., Abduro, K., & Chaka, L. (2015). Survey of gastrointestinal menatodes and anthelmintic resistance in sheep and goats in communal grazing pastoral area, Yabello District, southern Ethiopia. Ethiopian Veterinary Journal, 19(1), 35–47. https://doi.org/10.4314/evj.v19i1.5
57. Diriba, S., & Tulu, D. (2018). Prevalence of GIT parasites and associated risk factors in cattle of Lume, Bora, Dugda and Adamitulu Jido Kombalcha districts of East Shewa Zone. Academic Research Journal of Agricultural Science and Research, 6(5), 260–265. https://doi.org/10.14662/ARJASR2017.080
58. Dugassa, J., Hussein, A., Kebede, A., & Mohammed, C. (2018). Prevalence and associated risk factors of gastrointestinal nematodes of sheep and goats in ZiwayDugda District, Eastern Arsi Zone of Oromia Regional State, Ethiopia. ARC Journal of Animal and Veterinary Sciences, 4(2), 6–14. https://doi.org/10.20431/2455-2518.0402002
59. Dugassa, J., Hussein, A., Kebede, A., & Mohammed, C. (2018). Prevalence and associated risk factors of gastrointestinal nematodes of sheep and goats in ZiwayDugda District, Eastern Arsi Zone of Oromia Regional State, Ethiopia. ARC Journal of Animal and Veterinary Sciences, 4(2), 6–14. https://doi.org/10.20431/2455-2518.0402003
60. Tessema, W., Getachew, M., & Tora, E. (2024). Prevalence and risk factors of lungworm infection in small ruminants in selected districts of Wolaita Zone, Southern Ethiopia. Journal of Parasitology Research, 2024, Article ID 6303598. https://doi.org/10.1155/2024/6303598
61. Gobir, S. S., Suleiman, M., Sarkingobir, Y., Imam, A. U., Muhammed, S., & Sulaiman, B. (2023). Prevalence of zoonotic gastrointestinal parasite in cattle slaughtered at Gwadabawa abattoir of Sokoto State, Nigeria. CMU Journal of Science, 27(2), 80–85. https://doi.org/10.52751/CQYT6475
62. Greter, H., Batil, A. A., Ngandolo, B. N., Alfaroukh, I. O., Moto, D. D., Hattendorf, J., Utzinger, J., & Zinsstag, J. (2017). Human and livestock trematode infections in a mobile pastoralist setting at Lake Chad: Added value of a One Health approach beyond zoonotic diseases research. Transactions of the Royal Society of Tropical Medicine and Hygiene, 111(6), 278–284. https://doi.org/10.1093/trstmh/trx051
63. Haymanot, F., & Kaba, T. (2022). Prevalence and associated factors of gastrointestinal helminthiasis of lactating cows and effect of strategic deworming on milk quantity, fat, and protein in Kucha, Ethiopia. BMC Veterinary Research, 18, Article 150. https://doi.org/10.1186/s12917-022-03251-2
64. Ikpe, R. T., Agbendeh, N. L., & Akinsuyi, O. S. (2022). Prevalence study of zoonotic gastrointestinal parasitic infections in goats in Makurdi Metropolis, Nigeria. Journal of Zoonotic Diseases, 6(1), 11-16. https://doi.org/10.1001/1.2476535.2022.6.1.1
65. Jansen, M. S., Nyangiwe, N., Yawa, M., Dastile, M., Mabhece, V., Muchenje, V., … Mpendulo, T. C. (2020). Occurrence of gastrointestinal parasites in slaughter rabbits. Parasitology Research, 119(12), 4169–4175. https://doi.org/10.1007/s00436-020-06927-6
66. Kagenda, G. A., & Angwech, H. (2018). Cross-sectional prevalence of gastrointestinal helminth parasites in cattle in Lira District, Uganda. Tropical Animal Health and Production, 50(5), 1599–1604. https://doi.org/10.1007/s11250-018-1600-0
67. Kebede, I. A., Beriso, T. E., Mengistu, T. S., & Gebremeskel, H. F. (2023). Study on cattle trematodiasis and related risk factors in Damot Sore District, Wolaita Zone, Southern Ethiopia. Journal of Parasitology Research, 2023, Article ID 6687665. https://doi.org/10.1155/2023/6687665
68. Kipyegen, C. K., Muleke, C. I., & Otachi, E. O. (2022). Human and animal fasciolosis: Coprological survey in Narok, Baringo and Kisumu counties, Kenya. Onderstepoort Journal of Veterinary Research, 89(1), Article 1954. https://doi.org/10.4102/ojvr.v89i1.1954
69. Lakew, A., & Seyoum, Z. (2016). Ovine coccidiosis: Prevalence and associated risk factors in and around Addis-Zemen, Northwest Ethiopia. Turkish Journal of Veterinary & Animal Sciences, 40(5), 645–650. https://doi.org/10.3906/vet-1509-18
70. Mbong Erica Malla, Payne, V. K., Yamssi, C., Noumedem Anangmo, C. N., Megwi, L., & Matsinkou, R. (2021). Prevalence of gastrointestinal parasites in sheep and goats of Bui and Donga-Mantung Divisions of the North West Region of Cameroon. Asian Journal of Research in Animal and Veterinary Sciences, 4(2), 161–175. https://doi.org/10.9734/ajravs/2021/v4i2149
71. Mathewos, M., Endale, H., & Kebamo, M. (2023). Coprological and postmortem assessment and economic significance of bovine fasciolosis in cattle slaughtered at Tarcha Municipal Abattoir, Southern Ethiopia. Parasite Epidemiology and Control, 22, e00316. https://doi.org/10.1016/S2405-6731(23)00033-8
72. Tesfaye, M. G., Mideksa, M. T., Abera, H., Duguma, M., & Gudeta, T. (2016). Epidemiological study of gastro-intestinal helminthes parasites of calves in urban, peri-urban and rural smallholder dairy farms of East Wollega Zone, Western Ethiopia. Journal of Biology, Agriculture and Healthcare, 6(17), 29–34. https://www.researchgate.net/publication/367541761
73. Mochankana, M. E., & Robertson, I. D. (2018). Cross-sectional prevalence of Fasciola gigantica infections in beef cattle in Botswana. Tropical Animal Health and Production, 50(6), 1355–1363. https://doi.org/10.1007/s11250-018-1568-9
74. Mohammed, A., & Fasil, N. (2019). The prevalence of ovine gastrointestinal nematode and associated risk factor in Jigjiga Woreda, Fafan Zone, Somali Regional State, Eastern Ethiopia. Dairy and Veterinary Sciences Journal, 11(1), 555802. https://doi.org/10.19080/JDVS.2019.11.555802
75. Mohammed, N., Taye, M., Asha, A., & Sheferaw, D. (2016). Epizootological study of small ruminant gastrointestinal strongyles in Gamo-Gofa Zone, Southern Ethiopia. Journal of Parasitic Diseases, 40(2), 469–474. https://doi.org/10.1007/s12639-014-0528-1
76. Nana, T. (2016). Prevalence of ovine gastrointestinal nematodes in Meskan District, Gurage Zone, Southern Ethiopia. Journal of Natural Sciences Research, 6(15), 75–82.
77. Jahashi, N., Kassuku, A. A., Stothard, J. R., Coles, G. C., & Eisler, M. C. (2014). Trematode infections in cattle in Arumeru District, Tanzania are associated with irrigation. Parasites & Vectors, 7, 107. https://doi.org/10.1186/1756-3305-7-107
78. Oyewusi, I. K. (2017). Prevalence of gastrointestinal parasites of rams brought for slaughter at Bodija abattoir, Ibadan, Nigeria. Tropical Animal Health and Production. Advance online publication. https://www.ajol.info/index.php/tjas/article/view/163778
79. Thlama, P. B. (2016). Point prevalence and intensity of gastrointestinal parasite ova/oocyst and its association with body condition score (BCS) of sheep and goats in Maiduguri, Nigeria. The Journal of Advances in Parasitology, 3(3), 81–88. https://doi.org/10.14737/journal.jap/2016/3.3.81.88
80. Regasa, Z. A. (2018). Study on calf coccidiosis in dairy farms in and around Mekelle, Ethiopia. Journal of Veterinary Medicine and Animal Health, 10(6), 153–158. https://doi.org/10.5897/JVMAH2017.0631
81. Sabo, H., Quazim, J. O., Alkali, Y. A., Musa, A. G., & Yoriyo, K. P. (2023). Polyparasitism of gastrointestinal parasites among pastoral cattle herds and trade cattle in Gombe metropolis. Journal of Pure and Applied Sciences (Science Forum), 23(1), 21–28.
82. Seyoum, Z., Getnet, K., Chanie, M., Derso, S., & Fentahun, S. (2018). Morbidity parameters associated with gastrointestinal tract nematodes in sheep in Dabat District, Northwest Ethiopia. BioMed Research International, 2018, Article 9247439. https://doi.org/10.1155/2018/9247439
83. Sheferaw, D., Mohammed, A., & Degefu, A. (2021). Distribution and prevalence of gastrointestinal tract nematodes of sheep at highland and midland areas, Ethiopia. Journal of Parasitic Diseases, 45(4), 995–1001. https://doi.org/10.1007/s12639-021-01397-8
84. Shime, A. (2018). Helimenthe parasites of small ruminant in Gozamin Woreda. Journal of Natural Sciences Research, 8(17).
85. Oluwole, A. S., Adeniran, A. A., Mogaji, H. O., Shittu, E. O., Alabi, O. M., Odoemene, S., & Ekpo, U. F. (2016). Survey of gastrointestinal parasites among nomadic cattle herds in Eruwa, Oyo State, Southwestern Nigeria. Annual Research & Review in Biology, 10(6), 1–7. https://doi.org/10.9734/ARRB/2016/28400
86. Suleiman, M., Ahmed, A., & Gobir, S. S. (2022). Haemo and gastrointestinal parasites in cattle slaughtered at Gwadabawa abattoir in Sokoto State, north-western Nigeria. The Bioscientist, 10(1), 55–64.
87. Tesfaye, T. (2021). Prevalence, species composition, and associated risk factors of small ruminant gastrointestinal nematodes in South Omo Zone, South-western Ethiopia. Journal of Advanced Veterinary and Animal Research, 8(4), 597–605. https://doi.org/10.5455/javar.2021.h550
88. Teshome, D., Teferi, G., & Mengistu, Y. (2021). Epidemiological study of small ruminant gastrointestinal helminthosis in Borana Lowlands, Southern Oromia, Ethiopia. International Journal of Biomedical Engineering and Clinical Science, 7(3), 43–47. https://doi.org/10.11648/j.ijbecs.20210703.11
89. Tumusiime, M., Manishimwe, J. C., & Ntampaka, P. (2023). Prevalence of bovine trematodiases and associated risk factors in Nyagatare District, Rwanda. Veterinary Medicine: Research and Reports, 14, 221–231. https://doi.org/10.2147/VMRR.S430581
90. Unigwe, C. R., Balogun, F. A., Olona, J. F., Nwufoh, O. C., & Odah, I. S. (2017). Gastrointestinal helminths of West African Dwarf (WAD) goats in Ido Local Government Area, Ibadan, Oyo State, Nigeria. Journal of Animal Science and Veterinary Medicine, 2(4), 133–138. https://doi.org/10.31248/JASVM2016.019
91. Worku, K., Hamid, M., & Dubie, T. (2019). Study on prevalence and risk factors of calf coccidiosis in and around Sekota town, Northern Ethiopia. International Journal of Current Research in Biology and Medicine, 4(8), 7–16. https://doi.org/10.22192/ijcrbm.2019.04.08.002
92. Tadesse, Y., Mohammed, A., & Mekonnen, Z. (2016). Prevalence and risk factor identification of calf coccidiosis in and around Bahir Dar Town, Amhara Regional State, North-West Ethiopia. Journal of Animal Research, 6(5), 773–778. https://doi.org/10.5958/2277-940X.2016.00097.8
93. Karimou, I. A., et al. (2024). Prevalence and risk factors for gastrointestinal parasitosis in small ruminant farms in the Sahelian zone of Niger. Veterinary Parasitology: Regional Studies & Reports, 56, 101125. https://doi.org/10.1016/j.vprsr.2024.101125
94. Adelabu, D. B. (2016). Prevalence of gastro-intestinal parasites in small ruminants slaughtered at a selected slaughter slab in Ado Ekiti, Southwest Nigeria. Journal of Researches in Agricultural Sciences, 4(1–2), 5–5.
95. Agboola, L. L. (2013). Variability in prevalence and resistance to helminths infection in Nigerian sheep breeds as influenced by genetic and non-genetic factors. https://doi.org/10.13140/RG.2.2.20763.78887
96. Bedada, H., Gizaw, F., & Negash, W. (2017). Preliminary study on small ruminant GIT helminthiasis in select arid and semi-arid pastoral and agro-pastoral areas of Afar Region, Ethiopia. International Journal of Advanced Research in Biological Sciences, 4(12), 60–67. https://doi.org/10.22192/ijarbs.2017.04.12.006
97. Chalchisa, N., Yimer, M., & Bezabih, M. K. (2015). Prevalence of gastrointestinal strongyles parasites of small ruminants in and around Haramaya, Ethiopia. Middle East Journal of Scientific Research, 23(12), 2833–2840. https://doi.org/10.5829/idosi.mejsr.2015.23.12.101134
98. Dahourou, L. D., Konaté, A., Tapsoba, A. S. R., Dicko, A., Sanou, M., Tamboura, H. H., Bayala, B., Salissou, I., Traoré, A., Logan, L. L., & Tembely, S. (2021). Epidemiology and spatio-temporal distribution of gastrointestinal parasites infection and accuracy of FAMACHA test in sheep in traditional farming systems in Burkina Faso. Tropical Animal Health and Production, 53, 392. https://doi.org/10.1007/s11250-021-02810-3
99. Tefa, T., Ebissa, T., & Aki, A. (2018). Prevalence and risk factors of gastrointestinal nematodes infections in small ruminants in Sayyo District, Kellem Wollega Zone, Ethiopia. Report and Opinion, 10(4), 9–13. https://doi.org/10.7537/marsroj100418.02
100. Elelu, N., Ambali, A., Coles, G. C., & Eisler, M. C. (2016). Cross-sectional study of Fasciola gigantica and other trematode infections of cattle in Edu Local Government Area, Kwara State, north-central Nigeria. Parasites & Vectors, 9, Article 470. https://doi.org/10.1186/s13071-016-1737-5
101. Elemo, K. K., & Geresu, M. A. (2017). Prevalence and risk factors of gastrointestinal parasites of small ruminants in Sinana and Dinsho Districts of Bale Zone, South Eastern Ethiopia. European Journal of Biological Sciences, 9(1), 1–8. https://doi.org/10.5829/idosi.ejbs.2017.01.08
102. Farooq, U. B., Abdulla, A., Chaudhary, R., & Abera, M. (2018). A cross-sectional study on the prevalence and associated risk factors of gastrointestinal nematodes of sheep in and around Hirna, South East Ethiopia. International Journal of Livestock Research, 8(5), 89–97. https://doi.org/10.5455/ijlr.20170427114448
103. Gakuru, M. I. (2022). Prevalence of gastrointestinal parasites and associated factors in Zebu calves among pastoralists in Isiolo County, Kenya (Master’s thesis). University of Nairobi.
104. Getachew, M., Tesfaye, R., & Sisay, E. (2017). Prevalence and risk factors of gastrointestinal nematodes infections in small ruminants in Tullo District, Western Hararghe, Ethiopia. Journal of Veterinary Science & Technology, 8(2), Article 428. https://doi.org/10.4172/2157-7579.1000428
105. Husen, M., Aliyi, F., Damtew, S., Negassa, T., & Abebe, H. (2018). Prevalence of small ruminant helminthiases in and around Tullo District in Western Hararghe Zone, Eastern Ethiopia. Austin Journal of Veterinary Science & Animal Husbandry, 5(1), 1038.
106. Kalule, F., Vudriko, P., Nanteza, A., Ekiri, A. B., Alafiatayo, R., Betts, J., ... & Cook, A. (2023). Prevalence of gastrointestinal parasites and molecular identification of beta-tubulin mutations associated with benzimidazole resistance in Haemonchus contortus in goats from selected districts of Uganda. Veterinary parasitology: regional studies and reports, 42, 100889. https://doi.org/10.1016/j.vprsr.2023.100889
107. Mpofu, T. J., Nephawe, K. A., & Mtileni, B. (2020). Prevalence of gastrointestinal parasites in communal goats from different agro-ecological zones of South Africa. Veterinary World, 13(1), 26–32. https://doi.org/10.14202/vetworld.2020.26-32
108. Negash, B., Seyoum, W., & Sheferaw, D. (2023). Seasonal prevalence and status of anthelmintic resistance of goats’ gastrointestinal nematodes, Mirab Abaya, Southern Ethiopia. Journal of Parasitology Research, 2023, Article 9945998. https://doi.org/10.1155/2023/9945998
109. Ngetich, E. C., Ngeiywa, M., Ng’wena, G. M., & Chemwolo, L. (2019). Haematological parameters by age and sex of asymptomatic indigenous cattle and sheep infected with gastrointestinal parasites in Kerio Valley, Kenya. American Journal of Biomedical Research, 7(2), 44–50. https://doi.org/10.12691/ajbr-7-2-4
110. Ola-Fadunsin, S. D., Ganiyu, I. A., Rabiu, M., Hussain, K., Sanda, I. M., Baba, A. Y., Furo, N. A., & Balogun, R. B. (2020). Helminth infections of great concern among cattle in Nigeria: Insight to its prevalence, species diversity, patterns of infections and risk factors. Veterinary World, 13(2), 338–344. https://doi.org/10.14202/vetworld.2020.338-344
111. Squire, S. A., Yang, R., Robertson, I., Ayi, I., Squire, D. S., & Ryan, U. (2018). Gastrointestinal helminths in farmers and their ruminant livestock from the Coastal Savannah zone of Ghana. Parasitology Research, 117(10), 3183–3194. https://doi.org/10.1007/s00436-018-6017-1

**Supplementary Table 4.** Sensitivity testing (influence diagnostics) identifying highly influential studies affecting the pooled prevalence estimates of gastrointestinal parasite infection in cattle, sheep and goats in sub-Saharan Africa between 2015 and 2024

| **Study  ID** | **Authors** | **Studentized residuals** | **Cook's distance** | **Difference  in fits** | **Covariance ratio** | **Tau^2^ reduced** | **Hat value** | **Weight** | **Highly influential** |
| --- | --- | --- | --- | --- | --- | --- | --- | --- | --- |
| 1 | Tiele et al. (2023) | 0.34 | 0.00 | 0.03 | 1.02 | 0.06 | 0.01 | 0.91 | No |
| 2 | Tulu and Lelisa (2016) | -0.37 | 0.00 | -0.04 | 1.02 | 0.06 | 0.01 | 0.91 | No |
| 3 | Owusu et al. (2016) | 2.22 | 0.04 | 0.21 | 0.97 | 0.06 | 0.01 | 0.88 | **Yes** |
| 4 | Habtemichael et al. (2018) | -0.68 | 0.00 | -0.07 | 1.01 | 0.06 | 0.01 | 0.90 | No |
| 5 | Regea (2019) | -0.50 | 0.00 | -0.05 | 1.02 | 0.06 | 0.01 | 0.91 | No |
| 6 | Obi et al. (2020) | -0.06 | 0.00 | -0.01 | 1.02 | 0.06 | 0.01 | 0.90 | No |
| 7 | Bisimwa et al. (2018) | 0.64 | 0.00 | 0.06 | 1.01 | 0.06 | 0.01 | 0.90 | No |
| 8 | Mideksa et al. (2016) | 0.30 | 0.00 | 0.03 | 1.02 | 0.06 | 0.01 | 0.90 | No |
| 9 | Kagenda et al. (2017) | -1.11 | 0.01 | -0.11 | 1.01 | 0.06 | 0.01 | 0.90 | No |
| 10 | Dogo et al. (2017) | 0.45 | 0.00 | 0.04 | 1.02 | 0.06 | 0.01 | 0.91 | No |
| 11 | Umar et al. (2018) | 0.66 | 0.00 | 0.06 | 1.01 | 0.06 | 0.01 | 0.90 | No |
| 12 | Mpofu et al. (2020) | -0.90 | 0.01 | -0.09 | 1.01 | 0.06 | 0.01 | 0.91 | No |
| 13 | Chia et al. (2015) | -0.10 | 0.00 | -0.01 | 1.02 | 0.06 | 0.01 | 0.86 | No |
| 14 | Dabasa et al. (2017) | 0.83 | 0.01 | 0.08 | 1.01 | 0.06 | 0.01 | 0.91 | No |
| 15 | Habtemichael et al. (2018) | -0.68 | 0.00 | -0.07 | 1.01 | 0.06 | 0.01 | 0.90 | No |
| 16 | Muktar et al. (2015) | -0.65 | 0.00 | -0.06 | 1.01 | 0.06 | 0.01 | 0.91 | No |
| 17 | Lemy and Egwunyenga (2017) | -0.35 | 0.00 | -0.03 | 1.02 | 0.06 | 0.01 | 0.89 | No |
| 18 | Aliyu et al. (2020) | 0.80 | 0.01 | 0.08 | 1.01 | 0.06 | 0.01 | 0.90 | No |
| 19 | Eke et al. (2019) | 0.44 | 0.00 | 0.04 | 1.02 | 0.06 | 0.01 | 0.89 | No |
| 20 | Shime and Derso (2016) | 0.02 | 0.00 | 0.00 | 1.02 | 0.06 | 0.01 | 0.91 | No |
| 21 | Hussein et al. (2023) | -0.20 | 0.00 | -0.02 | 1.02 | 0.06 | 0.01 | 0.91 | No |
| 22 | Sebro et al. (2021) | 0.70 | 0.00 | 0.07 | 1.01 | 0.06 | 0.01 | 0.91 | No |
| 23 | Abdi-Soojeede (2018) | 0.54 | 0.00 | 0.05 | 1.02 | 0.06 | 0.01 | 0.87 | No |
| 24 | Mushonga et al. (2018) | 0.72 | 0.00 | 0.07 | 1.01 | 0.06 | 0.01 | 0.91 | No |
| 25 | Jaja et al. (2017) | -1.49 | 0.02 | -0.14 | 1.00 | 0.06 | 0.01 | 0.91 | No |
| 26 | Matsepe et al. (2021) | 1.91 | 0.03 | 0.18 | 0.99 | 0.06 | 0.01 | 0.91 | No |
| 27 | Walden et al. (2021) | 0.68 | 0.00 | 0.06 | 1.01 | 0.06 | 0.01 | 0.88 | No |
| 28 | Namutosi et al. (2019) | 0.65 | 0.00 | 0.06 | 1.01 | 0.06 | 0.01 | 0.88 | No |
| 29 | Mohamed et al. (2023) | 0.53 | 0.00 | 0.05 | 1.02 | 0.06 | 0.01 | 0.91 | No |
| 30 | Challaton et al. (2023) | 2.13 | 0.04 | 0.20 | 0.98 | 0.06 | 0.01 | 0.91 | **Yes** |
| 31 | Joan et al. (2015) | 0.45 | 0.00 | 0.04 | 1.02 | 0.06 | 0.01 | 0.91 | No |
| 32 | Mohammed et al. (2015) | 0.23 | 0.00 | 0.02 | 1.02 | 0.06 | 0.01 | 0.91 | No |
| 33 | Oyewusi et al. (2017) | -0.31 | 0.00 | -0.03 | 1.02 | 0.06 | 0.01 | 0.90 | No |
| 34 | Molla et al. (2023) | -0.19 | 0.00 | -0.02 | 1.02 | 0.06 | 0.01 | 0.91 | No |
| 35 | Jegede et al. (2015) | -0.77 | 0.01 | -0.07 | 1.01 | 0.06 | 0.01 | 0.88 | No |
| 36 | Moje et al. (2021) | 0.91 | 0.01 | 0.09 | 1.01 | 0.06 | 0.01 | 0.90 | No |
| 37 | Adua et al. (2017) | -0.31 | 0.00 | -0.03 | 1.02 | 0.06 | 0.01 | 0.86 | No |
| 38 | Kyari et al. (2023) | -1.31 | 0.01 | -0.12 | 1.00 | 0.06 | 0.01 | 0.86 | No |
| 39 | Abagero et al. (2023) | -1.09 | 0.01 | -0.10 | 1.01 | 0.06 | 0.01 | 0.91 | No |
| 40 | Abaya et al. (2023) | -1.33 | 0.02 | -0.13 | 1.00 | 0.06 | 0.01 | 0.91 | No |
| 41 | Abddulkadir et al. (2017) | -0.71 | 0.00 | -0.07 | 1.01 | 0.06 | 0.01 | 0.91 | No |
| 42 | Abebe et al. (2018) | -0.92 | 0.01 | -0.09 | 1.01 | 0.06 | 0.01 | 0.91 | No |
| 43 | Abera et al. (2019) | -1.48 | 0.02 | -0.14 | 1.00 | 0.06 | 0.01 | 0.91 | No |
| 44 | Adeyemi et al. (2021) | 0.69 | 0.00 | 0.07 | 1.01 | 0.06 | 0.01 | 0.89 | No |
| 45 | Agba et al. (2020) | -1.05 | 0.01 | -0.10 | 1.01 | 0.06 | 0.01 | 0.91 | No |
| 46 | Ahmed et al. (2017) | -0.09 | 0.00 | -0.01 | 1.02 | 0.06 | 0.01 | 0.91 | No |
| 47 | Alaku et al. (2021) | -0.40 | 0.00 | -0.04 | 1.02 | 0.06 | 0.01 | 0.89 | No |
| 48 | Alemayehu et al. (2021) | -1.05 | 0.01 | -0.10 | 1.01 | 0.06 | 0.01 | 0.91 | No |
| 49 | Alemnew et al. (2017) | 0.07 | 0.00 | 0.01 | 1.02 | 0.06 | 0.01 | 0.91 | No |
| 50 | Ashagrie et al. (2022) | -0.31 | 0.00 | -0.03 | 1.02 | 0.06 | 0.01 | 0.90 | No |
| 51 | Barre et al. (2023) | -0.77 | 0.01 | -0.07 | 1.01 | 0.06 | 0.01 | 0.89 | No |
| 52 | Belina et al. (2017) | -0.32 | 0.00 | -0.03 | 1.02 | 0.06 | 0.01 | 0.91 | No |
| 53 | Dawit et al. (2022) | -0.32 | 0.00 | -0.03 | 1.02 | 0.06 | 0.01 | 0.91 | No |
| 54 | Demissie et al. (2021) | 0.24 | 0.00 | 0.02 | 1.02 | 0.06 | 0.01 | 0.91 | No |
| 55 | Desalegn and Berhanu (2023) | 0.64 | 0.00 | 0.06 | 1.01 | 0.06 | 0.01 | 0.91 | No |
| 56 | Sheferaw et al. (2015) | 0.17 | 0.00 | 0.02 | 1.02 | 0.06 | 0.01 | 0.91 | No |
| 57 | Diriba and Tulu (2018) | -0.74 | 0.01 | -0.07 | 1.01 | 0.06 | 0.01 | 0.91 | No |
| 58 | Dugassa et al. (2018) | 0.54 | 0.00 | 0.05 | 1.02 | 0.06 | 0.01 | 0.91 | No |
| 59 | Dugassa et al. (2018) | -1.80 | 0.03 | -0.17 | 0.99 | 0.06 | 0.01 | 0.91 | No |
| 60 | Getachew (2022) | 1.70 | 0.03 | 0.16 | 0.99 | 0.06 | 0.01 | 0.88 | No |
| 61 | Gobir et al. (2023) | -2.23 | 0.04 | -0.21 | 0.97 | 0.06 | 0.01 | 0.88 | **Yes** |
| 62 | Greter et al. (2017) | -0.27 | 0.00 | -0.03 | 1.02 | 0.06 | 0.01 | 0.91 | No |
| 63 | Haymanot and Kaba (2022) | -0.97 | 0.01 | -0.09 | 1.01 | 0.06 | 0.01 | 0.91 | No |
| 64 | Ikpe et al. (2022) | 1.92 | 0.03 | 0.18 | 0.99 | 0.06 | 0.01 | 0.89 | No |
| 65 | Jansen et al. (2020) | 0.74 | 0.01 | 0.07 | 1.01 | 0.06 | 0.01 | 0.91 | No |
| 66 | Kagenda and Angwech (2018) | -1.11 | 0.01 | -0.11 | 1.01 | 0.06 | 0.01 | 0.90 | No |
| 67 | Kebede et al. (2023) | -1.39 | 0.02 | -0.13 | 1.00 | 0.06 | 0.01 | 0.91 | No |
| 68 | Kipyegen et al. (2022) | -1.17 | 0.01 | -0.11 | 1.01 | 0.06 | 0.01 | 0.91 | No |
| 69 | Lakew and Seyoum (2016) | -1.54 | 0.02 | -0.15 | 1.00 | 0.06 | 0.01 | 0.91 | No |
| 70 | Malla et al. (2021) | 1.49 | 0.02 | 0.14 | 1.00 | 0.06 | 0.01 | 0.91 | No |
| 71 | Mathewos et al. (2023) | -1.68 | 0.03 | -0.16 | 0.99 | 0.06 | 0.01 | 0.91 | No |
| 72 | Gelana et al. (2016) | 0.30 | 0.00 | 0.03 | 1.02 | 0.06 | 0.01 | 0.90 | No |
| 73 | Mochankana and Robertson (2018) | -3.39 | 0.10 | -0.33 | 0.92 | 0.05 | 0.01 | 0.92 | **Yes** |
| 74 | Mohammed and Fasil (2019) | 0.33 | 0.00 | 0.03 | 1.02 | 0.06 | 0.01 | 0.91 | No |
| 75 | Mohammed et al. (2016) | 0.47 | 0.00 | 0.04 | 1.02 | 0.06 | 0.01 | 0.91 | No |
| 76 | Nana (2016) | 0.06 | 0.00 | 0.01 | 1.02 | 0.06 | 0.01 | 0.90 | No |
| 77 | Nzalawahe et al. (2016) | 1.93 | 0.03 | 0.19 | 0.98 | 0.06 | 0.01 | 0.91 | No |
| 78 | Oyewusi et al. (2017) | -0.31 | 0.00 | -0.03 | 1.02 | 0.06 | 0.01 | 0.90 | No |
| 79 | Thlama et al. (2016) | 0.54 | 0.00 | 0.05 | 1.02 | 0.06 | 0.01 | 0.88 | No |
| 80 | Regasa et al. (2018) | -1.39 | 0.02 | -0.13 | 1.00 | 0.06 | 0.01 | 0.91 | No |
| 81 | Sabo et al. (2023) | 0.65 | 0.00 | 0.06 | 1.01 | 0.06 | 0.01 | 0.90 | No |
| 82 | Seyoum et al. (2018) | -0.07 | 0.00 | -0.01 | 1.02 | 0.06 | 0.01 | 0.89 | No |
| 83 | Sheferaw et al. (2021) | 1.10 | 0.01 | 0.11 | 1.01 | 0.06 | 0.01 | 0.91 | No |
| 84 | Shime et al. (2018) | 0.13 | 0.00 | 0.01 | 1.02 | 0.06 | 0.01 | 0.91 | No |
| 85 | Stephen et al. (2016) | 0.15 | 0.00 | 0.01 | 1.02 | 0.06 | 0.01 | 0.89 | No |
| 86 | Suleiman et al. (2023) | -0.97 | 0.01 | -0.09 | 1.01 | 0.06 | 0.01 | 0.88 | No |
| 87 | Tesfaye et al. (2021) | 0.56 | 0.00 | 0.05 | 1.02 | 0.06 | 0.01 | 0.90 | No |
| 88 | Teshome et al. (2021) | 0.19 | 0.00 | 0.02 | 1.02 | 0.06 | 0.01 | 0.91 | No |
| 89 | Tumusiime et al. (2023) | 0.40 | 0.00 | 0.04 | 1.02 | 0.06 | 0.01 | 0.88 | No |
| 90 | Unigwe et al. (2017) | 0.49 | 0.00 | 0.05 | 1.02 | 0.06 | 0.01 | 0.88 | No |
| 91 | Worku et al. (2019) | -1.18 | 0.01 | -0.11 | 1.01 | 0.06 | 0.01 | 0.91 | No |
| 92 | Yamral et al. (2019) | -1.75 | 0.03 | -0.17 | 0.99 | 0.06 | 0.01 | 0.91 | No |
| 93 | Karimou et al. (2024) | 1.92 | 0.03 | 0.18 | 0.99 | 0.06 | 0.01 | 0.91 | No |
| 94 | Adelabu (2016) | 0.19 | 0.00 | 0.02 | 1.02 | 0.06 | 0.01 | 0.88 | No |
| 95 | Agboola (2023) | 0.51 | 0.00 | 0.05 | 1.02 | 0.06 | 0.01 | 0.91 | No |
| 96 | Bedada et al. (2017) | 1.38 | 0.02 | 0.13 | 1.00 | 0.06 | 0.01 | 0.91 | No |
| 97 | Chalchisa et al. (2015) | 1.66 | 0.02 | 0.16 | 0.99 | 0.06 | 0.01 | 0.91 | No |
| 98 | Dahourou et al. (2021) | 1.60 | 0.02 | 0.15 | 0.99 | 0.06 | 0.01 | 0.91 | No |
| 99 | Itefa et al. (2018) | -0.34 | 0.00 | -0.03 | 1.02 | 0.06 | 0.01 | 0.91 | No |
| 100 | Elelu et al. (2016) | 0.84 | 0.01 | 0.08 | 1.01 | 0.06 | 0.01 | 0.91 | No |
| 101 | Elemo & Geresu (2017) | -0.26 | 0.00 | -0.02 | 1.02 | 0.06 | 0.01 | 0.91 | No |
| 102 | Farooq et al. (2018) | 0.50 | 0.00 | 0.05 | 1.02 | 0.06 | 0.01 | 0.91 | No |
| 103 | Gakuru (2022) | 0.33 | 0.00 | 0.03 | 1.02 | 0.06 | 0.01 | 0.91 | No |
| 104 | Getachew et al. (2017) | -0.34 | 0.00 | -0.03 | 1.02 | 0.06 | 0.01 | 0.91 | No |
| 105 | Husen et al. (2018) | -0.21 | 0.00 | -0.02 | 1.02 | 0.06 | 0.01 | 0.91 | No |
| 106 | Kalule et al. (2023) | 2.24 | 0.04 | 0.21 | 0.97 | 0.06 | 0.01 | 0.90 | **Yes** |
| 107 | Mpofu et al. (2020) | -0.90 | 0.01 | -0.09 | 1.01 | 0.06 | 0.01 | 0.90 | No |
| 108 | Negash et al. (2023) | -0.05 | 0.00 | 0.00 | 1.02 | 0.06 | 0.01 | 0.91 | No |
| 109 | Ngetich et al. (2019) | -0.12 | 0.00 | -0.01 | 1.02 | 0.06 | 0.01 | 0.90 | No |
| 110 | Ola-Fadunsin et al. (2020) | 0.93 | 0.01 | 0.09 | 1.01 | 0.06 | 0.01 | 0.91 | No |
| 111 | Squire et al. (2018) | 0.22 | 0.00 | 0.02 | 1.02 | 0.06 | 0.01 | 0.91 | No |
|  |  |  |  |  |  |  |  |  |  |

*Studentized residuals ≥ 2 were flagged as outlier studies and if the Cook’s D > median + 3 x interquartile range, the study was marked as influential (**yes**)

**Supplementary Table 5.** Sensitivity testing (influence diagnostics) of highly influential studies affecting the pooled prevalence estimates of gastrointestinal parasite infection in cattle, sheep, and goats across sub-Saharan Africa between 2015 and 2024

| **Study  ID** | **Species** | | **Authors** | **Studentized residuals** | **Cook's distance** | | **Difference  in fits** | | **Covariance ratio** | **Tau^2^ reduced** | **Hat value** | **Weight** |
| --- | --- | --- | --- | --- | --- | --- | --- | --- | --- | --- | --- | --- |
| 73 | Cattle | Mochankana and Robertson (2018) | | -3.20 | | 0.19 | -0.48 | 0.85 | | 0.05 | 0.02 | 2.22 |
| 77 | Cattle | Nzalawahe et al. (2016) | | 2.61 | | 0.14 | 0.39 | 0.91 | | 0.05 | 0.02 | 2.20 |
| 3 | Sheep | Owusu et al. (2016) | | 2.50 | | 0.10 | 0.34 | 0.93 | | 0.04 | 0.02 | 1.77 |
| 38 | Sheep | Kyari et al. (2023) | | -2.09 | | 0.07 | -0.26 | 0.96 | | 0.04 | 0.02 | 1.56 |
| 59 | Sheep | Dugassa et al. (2018) | | -2.53 | | 0.11 | -0.35 | 0.92 | | 0.04 | 0.02 | 1.84 |
| 69 | Sheep | Lakew and Seyoum (2016) | | -2.18 | | 0.08 | -0.30 | 0.95 | | 0.04 | 0.02 | 1.84 |
| 97 | Sheep | Chalchisa et al. (2015) | | 1.96 | | 0.07 | 0.27 | 0.97 | | 0.04 | 0.02 | 1.81 |
| 30 | Goats | Challaton et al. (2023) | | 1.98 | | 0.08 | 0.29 | 0.96 | | 0.06 | 0.02 | 2.15 |
| 40 | Goats | Abaya et al. (2023) | | -2.73 | | 0.14 | -0.39 | 0.90 | | 0.05 | 0.02 | 1.99 |
| 106 | Goats | Kalule et al. (2023) | | 2.10 | | 0.09 | 0.31 | 0.95 | | 0.06 | 0.02 | 2.11 |

*The influential studies shown in this table were flagged when studentized residuals were ≥ 2 and the Cook’s D was > median + 3 x interquartile range.

**Supplementary Figure 1.** *Frequency of gastrointestinal parasite genera reported in (A) cattle (B) Sheep, and (C) goat fecal samples across sub-Saharan Africa between 2015 and 2024*
